# Design and Experimental Application of a Novel Non-Degenerate Universal Primer Set that Amplifies Prokaryotic 16S rRNA Genes with a Low Possibility to Amplify Eukaryotic rRNA Genes

**DOI:** 10.1093/dnares/dst052

**Published:** 2013-11-25

**Authors:** Hiroshi Mori, Fumito Maruyama, Hiromi Kato, Atsushi Toyoda, Ayumi Dozono, Yoshiyuki Ohtsubo, Yuji Nagata, Asao Fujiyama, Masataka Tsuda, Ken Kurokawa

**Affiliations:** 1 Department of Biological Information, Graduate School of Bioscience and Biotechnology, Tokyo Institute of Technology, 4259 B-36, Nagatsuta-cho, Midori-ku, Yokohama 226-8501, Japan; 2 Earth-Life Science Institute, Tokyo Institute of Technology, 2-12-1-E3-10 Ookayama, Meguro-ku, Tokyo 152-8550, Japan; 3 Section of Bacterial Pathogenesis, Graduate School of Medical and Dental Sciences, Tokyo Medical and Dental University, 1-5-45, Yushima, Bunkyo-Ku, Tokyo 113-8510, Japan; 4 Graduate School of Life Sciences, Tohoku University, 2-1-1 Katahira, Sendai 980-8577, Japan; 5 Center for Information Biology, National Institute of Genetics, Shizuoka 411-8540, Japan; 6 Principles of Informatics Research Division, National Institute of Informatics, Hitotsubashi, Chiyoda-ku, Tokyo 101-8430, Japan

**Keywords:** 16S rRNA, primer design, non-degenerate primer, microbial community

## Abstract

The deep sequencing of 16S rRNA genes amplified by universal primers has revolutionized our understanding of microbial communities by allowing the characterization of the diversity of the uncultured majority. However, some universal primers also amplify eukaryotic rRNA genes, leading to a decrease in the efficiency of sequencing of prokaryotic 16S rRNA genes with possible mischaracterization of the diversity in the microbial community. In this study, we compared 16S rRNA gene sequences from genome-sequenced strains and identified candidates for non-degenerate universal primers that could be used for the amplification of prokaryotic 16S rRNA genes. The 50 identified candidates were investigated to calculate their coverage for prokaryotic and eukaryotic rRNA genes, including those from uncultured taxa and eukaryotic organelles, and a novel universal primer set, 342F-806R, covering many prokaryotic, but not eukaryotic, rRNA genes was identified. This primer set was validated by the amplification of 16S rRNA genes from a soil metagenomic sample and subsequent pyrosequencing using the Roche 454 platform. The same sample was also used for pyrosequencing of the amplicons by employing a commonly used primer set, 338F-533R, and for shotgun metagenomic sequencing using the Illumina platform. Our comparison of the taxonomic compositions inferred by the three sequencing experiments indicated that the non-degenerate 342F-806R primer set can characterize the taxonomic composition of the microbial community without substantial bias, and is highly expected to be applicable to the analysis of a wide variety of microbial communities.

## 1. Introduction

Culture-independent and 16S rRNA gene-based analysis is widely used to profile microbial communities.^1^ Such profiling of 16S rRNA genes has revolutionized our understanding of microbial communities by allowing the characterization of the diversity of the uncultured majority.^2^ Moreover, ongoing development of massively parallel DNA sequencing technologies allows deep sequencing of a sample and simultaneous sequencing of many samples per run via low-cost bar-coding techniques.^3^ Because of its advantages over the traditional Sanger sequencing, the massively parallel sequencing has been applied to large-scale sequencing projects, including the analysis of 16S rRNA gene amplicons from diverse microbial communities.^4,5^

The Roche 454 Genome Sequencer FLX system^6^ is commonly used for the massively parallel sequencing of the 16S rRNA gene amplicons.^5^ Several studies have suggested that short (450–600 nt) reads obtained by the 454 platform^7^ are sufficient for the taxonomic assignment and the subsequent community comparison when the regions in 16S rRNA genes to be sequenced are carefully chosen.^8^ Notably, appropriate primer selection is a critical issue for analysing diverse microbial communities.^9^ The bias arising from poor complementarity between primers and 16S rRNA genes in particular taxa leads to underestimation of these taxa in a microbial community.^10,11^ Although the primer sets specific to *Bacteria* (e.g. the 338F and 533R set, which PCR-amplifies and covers the V3 regions of 16S rRNA genes)^12^ have generally been used for community profiling,^5,12^ recent studies of *Archaea* have revealed its ubiquitous distribution in diverse environments and its important roles in various ecosystems (e.g. ammonia oxidization in soil and seawater).^13^ To investigate the relative composition of *Bacteria* and *Archaea* and their ecological interaction in a microbial community,^14^ universal primer sets that can amplify both bacterial and archaeal 16S rRNA genes are needed.^15^ Although many sets of universal primers have been designed for this purpose,^9,10,15–17^ some such primers have led to the amplification of eukaryotic 18S rRNA genes and small-subunit rRNA genes from mitochondria and chloroplasts.^10,18^ Moreover, it is often difficult to estimate the number and diversity of the eukaryotic rRNA genes present in the environment before conducting the sequence-based community analysis.^18^ Therefore, the use of primers that amplify rRNA genes of eukaryotic origins has led to mischaracterization of the diversity of microbial communities that were associated with eukaryotic cells.^19,20^ This unfavourable situation is very serious in the case of host-associated habitats (e.g. the human skin, oral cavity, and vagina).^21^

The aim of this study is to overcome the problems associated with primer choice, and we attempted to identify the novel universal primers that complement only conserved bacterial and archaeal 16S rRNA gene sequences so that eukaryotic and organelle rRNA gene sequences would not be amplified. In general, the commonly used degenerate primers do not always achieve a better taxonomic coverage than the non-degenerate primers,^22^ and other recently designed degenerate primers still introduce a level of bias into the community profile.^23^ Therefore, our present study was focused on only the non-degenerate universal primers. The primer set identified in this study was experimentally used to show the taxonomic composition of a soil microbial community by amplifying 16S rRNA genes from the microbial metagenomic sample and subsequent pyrosequencing using the Roche 454 system. To evaluate the appropriateness of our original primer set to the taxonomic bias that might result from the PCR amplification, the same metagenomic sample was also used for (i) pyrosequencing of the 16S rRNA gene fragments after their amplification by use of the 338F and 533R set and (ii) the Illumina Genome Analyzer IIx (GA IIx) platform-based shotgun^24^ sequencing of the metagenome and subsequent finding and analysis of the 16S rRNA gene fragments, and the taxonomic compositions inferred by the three sequencing experiments were compared.

## 2. Materials and methods

### 2.1 Identification of universal primer candidates from genome-sequenced strains

To identify the candidate primer sequences for the PCR amplification of prokaryotic 16S rRNA genes, such genes from genome-sequenced strains were used as references because they have been accurately sequenced, are full-length genes, have well-defined taxonomic information. Bacterial and archaeal genomic sequences were obtained from the NCBI Genome Database (ftp://ftp.ncbi.nih.gov/genbank/genomes/Bacteria/, accessed on 11 November 2013) in November 2008. The 16S rRNA gene sequences in each strain were identified by RNAmmer.^25^ Then, one 16S rRNA gene sequence per species was randomly chosen because slight sequence differences exist among the 16S rRNA genes from strains of the same species,^26^ and among the gene copies within a genome.^27^ A total of 531 16S rRNA gene sequences were chosen. Their taxonomic information was obtained from the NCBI Taxonomy Database (http://www.ncbi.nlm.nih.gov/Taxonomy/taxonomyhome.html/, accessed on 11 November 2013). A multiple sequence alignment of the 531 16S rRNA gene sequences was constructed using MAFFT version 6.713 with default parameters.^28^

To find out the candidate sequences described above, highly conserved regions identified in the reference alignment were chosen as follows. Generally, the primer lengths for the PCR-amplification of 16S rRNA genes are more than 15 nt;^9^ therefore, we used a sliding window of 15 nt with a step size of 1 nt across the reference alignment. For each window, we calculated the frequency of each 15-nt sequence with one mismatch allowed. The 15-nt sequences that included gaps were also considered when calculating the frequencies. The consensus sequence for each window was defined as the 15-nt sequence that was found most frequently within one mismatch among strains. The coverage rate for a consensus sequence in each phylum was defined as the percentage of matched sequences among genome-sequenced strains within one mismatch.

### 2.2 Evaluation of each candidate universal sequence using the databases of the Ribosomal Database Project and the comparative RNA Web site

Each candidate sequence was evaluated using the Probe Match program in the Ribosomal Database Project (RDP) (release 10, update 22; http://rdp.cme.msu.edu/probematch/search.jsp, accessed on 11 November 2013).^29^ The 615 916 16S rRNA gene sequences in the RDP were retrieved by using the parameters Strain = Both, Source = Both, Size ≥1200, and Quality = Good, and then these sequences were used as reference sequences of the Probe Match program for the evaluation of the candidate sequences. The coverage for a candidate sequence in each phylum of the reference sequences was calculated as the percentage of genera for which more than half of the member sequences in each genus were found to match the candidate sequence within one mismatch. Because the reference sequences contain many uncultured strains,^29^ their taxonomic classifications are ambiguous, especially at the species level (e.g. description of the sp. as the taxa). Therefore, we used the genus, but not the species, to calculate the coverage for each candidate sequence. More than half of the 16S rRNA gene sequences in the reference sequences lacked their 5′- or 3′-terminal regions at the time of our analysis (http://rdp.cme.msu.edu/download/release10_22_containPosition.xls, accessed on 11 November 2013). Therefore, the candidate se-quences corresponding to such regions were excluded from the subsequent analyses.

The possibility of amplifying eukaryotic and organelle rRNA genes was evaluated by calculating the coverage for each primer candidate and the eukaryotic and organelle rRNA genes that were obtained from the comparative RNA Web (CRW).^30^ Aligned rRNA sequences of chloroplasts, mitochondria, and eukaryotes were separately downloaded from the CRW (http://www.rna.ccbb.utexas.edu/DAT/3A/Summary/index.php, accessed on 11 November 2013). The sequences containing ambiguous nucleotides (e.g. any nucleotide, N, or pyrimidine, Y) were discarded. One 16S rRNA sequence per genus was randomly chosen to reduce taxonomic bias in the dataset. After the conversion of uracil residues to thymine ones in the sequences, the coverage for each candidate sequence in the CRW-derived and subsequently processed sequences (76 chloroplastic, 480 mitochondrial, and 583 eukaryotic ones) was calculated as the percentage of matched sequences within two mismatches to eliminate the possible amplification of such rRNA genes. Based on the results of prior studies,^18^ we assumed that three or more mismatches between a primer and template would not allow the successful PCR amplification. The candidate sequences that covered more than 50% of the mitochondrial and eukaryotic rRNA gene sequences were also excluded to avoid the amplification of such genes. To reduce the possibility of random and non-specific amplification of non-rRNA genes, we merged neighbouring candidate sequences to extend their sequence lengths while maintaining the coverage for each phylum.

The validity of our primers was compared with the validity of 17 other universal primers published in the literature (Supplementary Table S1).^9,10,12,31^ We investigated the coverage of our and the 17 other primer sequences for (i) the 16S rRNA genes from bacterial and archaeal phyla in the reference sequences and (ii) the CRW-derived chloroplastic, mitochondrial, and eukaryotic rRNA genes described above. To avoid the difficulties of *in silico* evaluation, all ambiguous nucleotides in the 17 primers were converted to the nucleotides that gave rise to the best coverage for the reference sequences of bacterial and archaeal origins.

### 2.3 Sample processing and sequencing of a soil microbial community

A brown forest soil sample was collected in April 2008 from farmland at the Ehime Research Institute of Agriculture, Forestry, and Fisheries, Matsuyama, Japan,^32^ sieved through a 2-mm mesh to remove larger particles, and transferred to a sterilized glass pot with a loose lid. After its water content was adjusted to 60% of the maximum water-holding capacity, the soil was incubated at 28°C for 24 weeks. Total DNA was thereafter extracted from 10 g of soil using a PowerMax Soil DNA Isolation kit (MoBio, Carlsbad, CA, USA) according to the manufacturer's instructions. The DNA sample was concentrated by ethanol precipitation to obtain sufficient amount of DNA for PCR and metagenomic sequencing. PCR amplification of 16S rRNA genes was performed in 50 µl of 1 × *Ex Taq* buffer (2 mM Mg^2+^ Plus; Takara Bio, Ohtsu, Japan) containing 0.2 mM dNTPs, 0.625 U of TaKaRa *Ex Taq* HS (Takara Bio), 0.2 µM each of the forward and reverse primers, 2% dimethylsulphoxide, 0.01% bovine serum albumin, and 30 ng template DNA. Two PCR primer sets, 342F-806R and 338F-533R,^12^ were used. The PCR programme consisted of a single cycle of 95°C for 30 s, followed by 25 cycles of 95°C for 30 s, 55°C for 30 s, and 72°C for 15 s. A final extension was performed at 72°C for 7 min. PCR amplicons from eight parallel reactions were individually concentrated and purified by gel electrophoresis, and subsequently extracted to obtain more than 2 µg of amplicons. Pooled amplicons were sequenced on a 454 GS FLX Titanium one-sixteenth picotiter plate according to the manufacturer's protocols. In addition, 1-µg portions of metagenomic DNA were sheared to approximately 200-bp fragments using a Covaris-S instrument (Covaris, Woburn, MY, USA), and adapter sequences were ligated to both ends of the DNA fragments to generate a paired-end library. The library was subjected to 76 cycles of paired-end sequencing with an Illumina GA IIx instrument (one lane of an eight-lane flow cell) using reagents of an Illumina Sequencing kit, version 3.0, followed by base-calling using GA pipeline, version 1.5.0.

### 2.4 Sequence analysis

We used the high-quality 454 platform reads after removal of the reads that (i) contained ambiguous nucleotides, (ii) contained <150 or >250 nt in the 338F-533R experiment and <350 or >550 nt in the 342F-806R experiment, and (iii) were associated with an average Phred-like quality score of less than 20 as calculated by the 454 sequencer. Both the forward and reverse primer sequences were removed using cross-match, version 1.090518 (-minmatch 10 -minscore 12) (http://www.phrap.org/phredphrapconsed.html, accessed on 11 November 2013). Reverse-complemented reads were converted so that all reads started according to standard-sense strand conventions. Chimera sequences were detected and removed by the ChimeraSlayer in mothur version 1.11,^33^ using the Silva reference alignment (http://www.mothur.org/w/images/f/f1/Silva.gold.bacteria.zip, accessed on 11 November 2013). We also discarded the Illumina reads that (i) were evaluated as quality ‘N’ by purity filtering using the GA pipeline version 1.5.0 and (ii) contained more than one nucleotide with the quality-flag ‘B’ in the first 60 nt.

From the 615 916 16S rRNA gene sequences that were used as the reference sequences (see above), we eliminated (i) the 117 059 sequences whose origins at the genus-level were designated as ‘unclassified,’ and (ii) forty 23S rRNA gene-derived sequences with lengths longer than 2000 nt. Six sequences (S001337843, S001337931, S001337937, S001338015, S001338173, and S001338177) that contained unusually long homopolymers were also discarded because such homopolymers have returned many hits in our sequence similarity searches of the Illumina reads. Consequently, we obtained a database consisting of a total of 498 811 high-quality 16S rRNA gene sequences whose origins at the phylum and genus are definite, and we designated this database the ‘in-house high-quality RDP’ database. This database for *Bacteria* and *Archaea* was merged with the CRW-derived and above-described rRNA gene sequence database for chloroplasts, mitochondria, and eukaryotes.

Taxonomic assignment of the high-quality reads that were obtained from the 454 amplicon sequencing by use of the 338F-533R and 342F-806R primer sets was performed by a BLASTN^34^ search [e-value <1e−8; Identity ≥94% (genus) or ≥85% (phylum); and Alignment length ≥100 (for 338F-533R) or ≥300 (for 342F-806R)] against our merged database described above. The thresholds for alignment length were necessary to eliminate inaccurate or false-positive alignments. Identification and taxonomic assignment of 16S or 18S rRNA gene fragments in the reads obtained by the Illumina metagenomic sequencing was performed by a BLASTN search [e-value <1e−5; Identity ≥94% (genus) or ≥85% (phylum); and Alignment length ≥50] of our merged database.

When a best hit with the largest BLAST bit score existed, one was added to the taxon of the best hit for calculation of the taxonomic composition of the microbial community. When two or more database sequences had the same best BLAST bit score, one divided by the number of best hits was added to the corresponding taxa of the best hits. To improve the accuracy of the taxonomic assignments of the Illumina reads, we used only the read pairs whose sequences (i) were identified as those in a 16S rRNA gene from the same taxon and (ii) had a consistent alignment direction between the paired reads. The distance between the paired reads was not considered because the 16S rRNA genes in some taxa contain long insertions.^35^

### 2.5 Nucleotide sequence accession numbers

Sequences have been submitted to the DDBJ Sequence Read Archive under the following accession numbers: DRR001479 and DRR001475 for the 454-read data obtained using the 338F-533R and 342F-806R primer sets, respectively, and DRR001464 for the Illumina read data by metagenomic sequencing.

## 3. Results and discussion

### 3.1 Identification of universal primer candidates for 16S rRNA genes from genome-sequenced strains

There is a large collection of 16S rRNA gene sequences in the databases,^26,29,30^ and almost all such sequences were obtained by use of PCR. Therefore, the sequences that failed to match the universal primers do not exist in the database.^36^ Furthermore, many sequences in the database still contain the primer sequences used for PCR.^37^ On the other hand, the 16S rRNA gene sequences from the genome-sequenced prokaryotic strains contain the complete 16S rRNA gene sequences, including both terminal regions, which are missing in many cases in the databases (e.g. see http://rdp.cme.msu.edu/download/release10_22_containPosition.xls, accessed on 11 November 2013). Therefore, the 16S rRNA gene sequences from the genome-sequenced strains are expected to provide us accurate information on the variability of 16S rRNA genes from many different taxa.

**Figure 1.**
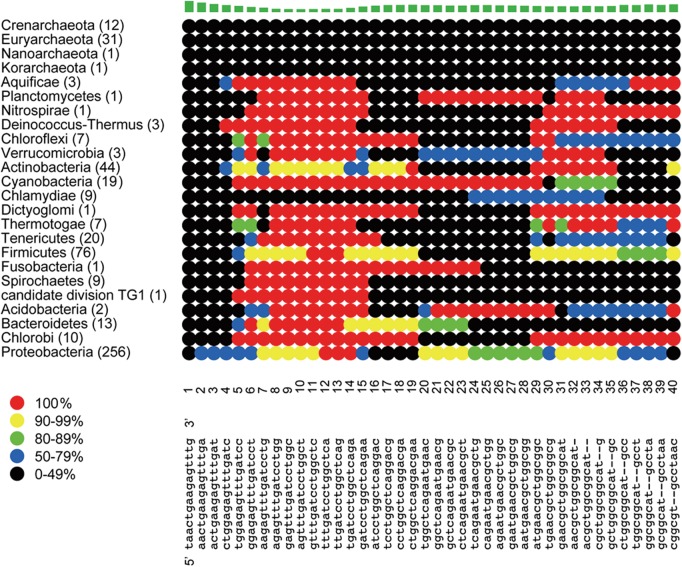
A partial plot of the consensus sequence in each window and its coverage rate for each phylum from 531 16S rRNA genes of genome-sequenced strains. The number of species in each phylum is indicated in parentheses. The start position of each window is represented according to the corresponding position in the 16S rRNA gene from *Escherichia coli*. The consensus sequence in each window is represented at the bottom of the figure. Each line indicate the coverage rate for the consensus sequence of each phylum by using the colours of dots: black <50%, blue <80%, green <90%, yellow <100%, and red = 100%. The bar graph at the top of the figure indicates the sequence variability of each window that is depicted by (i) calculating the relative entropy of four nucleotides and the gap (−) at each site^38^ and (ii) summing up the relative entropy of each site in the window.

Information concerning the consensus sequence for each 15-nt window and its coverage rate for 16S rRNA genes from the genome-sequenced strains of each phylum is shown in Figure 1 (some of the windows) and Supplementary Figure S1 (all windows). The windows in which almost all of the dots for the archaeal (top four) and bacterial lines showed >90% sequence identities (coloured red or yellow) were the universal primer candidate sequences. The candidates with conserved sequences for almost all bacterial and archaeal phyla were identified, and 50 such sequences are listed in Supplementary Table S2. Although the list was prepared based on the genomic data available in November 2008, the use of the updated data in July 2013 did not affect the conservation pattern of 50 universal primer candidate sequences across phyla (Supplementary Table S3). Our 50 universal primer candidate sequences are well overlapped with the degenerate primer candidates proposed by Wang and Qian (Supplementary Table S4),^9^ in spite that their algorism and database used were different from ours. Since the maximum sequencing read by the 2nd-generation sequencers at the time of our study was up to 450 nt, 17 out of the 50 candidate universal primer sequences that are located at the 3′-terminal regions of 16S rRNA genes (e.g. sequences 1390–1395, 1491–1496, and 1525–1526, Supplementary Fig. S1) were excluded for further analysis. Because the latest systems allow the determination of sequences up to 1100 nt in length (e.g. the PacBio sequencer^7^ and 454 GS FLX+ system; http://454.com/products/gs-flx-system/index.asp, accessed on 11 November 2013), the excluded sequences might be useful as primer candidates in the near feature.

### 3.2 Coverage for our candidate primer sequences and other universal primer sequences in bacterial, archaeal, eukaryotic, and organelle rRNA genes

We calculated the coverage for each of the remaining 33 candidates for rRNA genes from each bacterial and archaeal phylum in our in-house high-quality RDP database and from chloroplasts, mitochondria, and eukaryotes in the CRW-derived and above-described database, and the results are depicted as a heat map (Supplementary Fig. S2). All of the candidate sequences covered most of the chloroplastic rRNA genes within two mismatches, indicating the difficulty in avoiding their amplification during microbial community profiling. Twenty-one out of the 33 primer candidates [514–522 (nine candidates), 781–788 (seven candidates), and 907–915 (five candidates)] had relatively large coverage in bacterial and archaeal 16S rRNA genes. However, these 21 candidate sequences also covered the mitochondrial and eukaryotic rRNA genes within two mismatches; therefore, these sequences were not further considered as candidates. In cases in which the targeted microbial community contains only a small fraction of *Eukaryota*, these primers might be a good choice for the community profiling.

The remaining 12 candidates [341–343 (three candidates), 789–792 (four candidates), 879–880 (two candidates), and 1060–1066 (three candidates)] had sequences that appeared to exclude possible amplification of mitochondrial and eukaryotic rRNA genes. Because (i) the maximum length of a high-quality read for the 454 GS FLX Titanium system was ∼450 nt and (ii) the longer sequences provide more accurate taxonomic assignments,^8^ we chose candidates 341–343 and 789–792 as forward and reverse universal primer candidates, respectively. After merging the sequences of the neighbouring candidates, but maintaining the coverage in each phylum, we finally chose the sequence from 342 to 357 (5′-CTACGGGGGGCAGCAG-3′; 16 nt: 342F) and that from 790 to 806 (5′-GGACTACCGGGGTATCT-3′; 17 nt: 806R) as potential forward and reverse universal primers, respectively.

**Figure 2.**
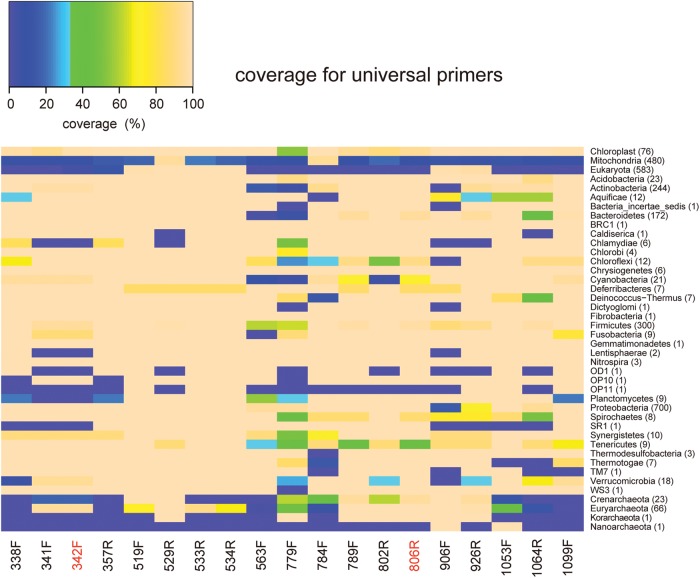
The coverage for our universal primer set (red) and the 17 other universal primers in each of the bacterial and archaeal phyla rRNA genes, and the chloroplastic, mitochondrial, and eukaryotic rRNA genes. Each column indicates the coverage for a candidate sequence from each of the bacterial and archaeal phyla rRNA genes, and the chloroplastic, mitochondrial, and eukaryotic rRNA genes by colour intensity: blue = low coverage, and pale orange = high coverage. See Supplementary Table S1 for each sequence. The number of genera is provided in parentheses.

The coverage for our universal primer set and the 17 other universal primers (Supplementary Table S1) in each of the bacterial and archaeal phyla rRNA genes, and the chloroplastic, mitochondrial, and eukaryotic rRNA genes is represented as a heat map (Fig. 2). The previously designed primers,^9,10,12,31^ 519F–534R (four primers), 784F, 906F, and 926R, covered mitochondrial and/or eukaryotic rRNA genes within two mismatches. The coverage for our universal primer set was, when compared with those for the 17 other universal primers, relatively large for bacterial and archaeal rRNA genes; however, relatively poor coverage was observed for some phyla (e.g. *Crenarchaeota*, *Planctomycetes*, OD1, and *Chlamydiae*). Furthermore, our universal primers had four mismatches (on average) against mitochondrial and eukaryotic rRNA genes. Most of the 19 primers covered chloroplastic rRNA genes within two mismatches (Supplementary Table S5). This result and the data in Supplementary Fig. S2 indicate that all of universal primer sequences examined in this study would be difficult to avoid the amplifications of chloroplastic rRNA genes. Finding a universal primer that avoids the amplification of chloroplastic rRNA genes is an important issue to be carried out in the future.

In general, the broad-range primers targeting bacterial and archaeal 16S rRNA genes tended to also amplify eukaryotic rRNA genes.^10,18,20^ When researchers analyse the microbial communities that contain many eukaryotic cells, the use of broad-range primers will lead to a decrease in the efficiency of sequencing and the mischaracterization of the microbial diversity in each community.^19^ Such communities are exemplified by eukaryotic host-associated ones: for example, the human microbiome project revealed that the average ratios of human DNA in the total metagenomic DNA extracted from several human body sites were about 50%.^21^ A broad-range primer, 515F, is used as a standard universal primer to analyse microbial communities in several environments in the Earth Microbiome Project (http://www.earthmicrobiome.org/, accessed on 11 November 2013). Since the 515F primer perfectly matches to many eukaryotic, mitochondrial, and chloroplastic rRNA genes, some studies that analysed animal-associated environments reported that the use of this primer also led to the amplification of many eukaryotic, mitochondrial, and chloroplastic rRNA genes.^39^ We primarily aimed to identify novel non-degenerate universal primers that were expected to amplify most bacterial and archaeal rRNA genes, but not eukaryotic rRNA genes. Because of this aim, our novel universal primers could not easily amplify the 16S rRNA genes from some bacterial and archaeal taxa without bias. Examples of such underrepresented taxa are the archaeal phyla, *Crenarchaeota*, *Korarchaeota,* and *Nanoarchaeota*, and the bacterial phyla, *Chlamydiae*, *Lentisphaerae*, OD1, OP11, *Planctomycetes,* and SR1 (Fig. 2). If these taxa are the major members in a microbial community of an environment, use of the 342F-806R primer set for the amplicon sequencing analysis will give rise to significantly biased taxonomic composition. To avoid misunderstanding of the compositions, the prior information related to the taxonomic composition of the community is very important. For example, *Crenarchaeota* is a major archaeal phylum;^40^ thus, in the analysis of the microbial community rich in this phylum, the combined use of our universal primer set with an established *Archaea*-specific universal primer set^10,17^ will allow more accurate description of the composition of the community. Since both of the primer sets can amplify 16S rRNA genes from *Euryarchaeota*, the relative abundance of this phylum in the community can be inferred, allowing us to calibrate the relative abundance of *Crenarchaeota* to that of *Bacteria* in the community.

### 3.3 Comparison of soil microbial community profiling by pyrosequencing with the two sets of primer pairs and by Illumina metagenomic sequencing

Experimental validation of our universal primers was conducted by 16S rRNA gene-based profiling of a soil microbial community with the 454 pyrosequencing. To assess whether our primer set enabled us to amplify most bacterial taxa in the soil sample in comparison with other primer sets, a commonly used bacteria-specific primer set, 338F-533R,^12^ was also used for the 454 pyrosequencing. The use of 16S rRNA gene fragments obtained by shotgun metagenomic sequencing can eliminate any bias that might be associated with the initial PCR amplification of 16S rRNA genes for the pyrosequencing.^41^ Therefore, metagenomic sequencing was additionally performed using the Illumina platform with paired-end libraries of the same sample to examine whether use of our universal primers resulted in taxonomic bias. Our sequencing and subsequent filtering generated 15 165 (338F-533R) and 27 527 (342F-806R) high-quality 454 reads by 16S rRNA gene amplicon sequencing, and 12 825 888 high-quality Illumina reads (6 412 944 read pairs) by metagenomic sequencing. A total of 3026 Illumina reads were assigned to 16S rRNA genes.

Our use of the two primer sets generated no 454 reads that were assigned to mitochondrial or eukaryotic rRNA genes from our refined CRW database (see Materials and methods), although eight Illumina read pairs were assigned to 18S rRNA genes from some fungi. Therefore, our universal primers did not allow the detection of the amplification of mitochondrial or eukaryotic rRNA genes.

**Figure 3.**
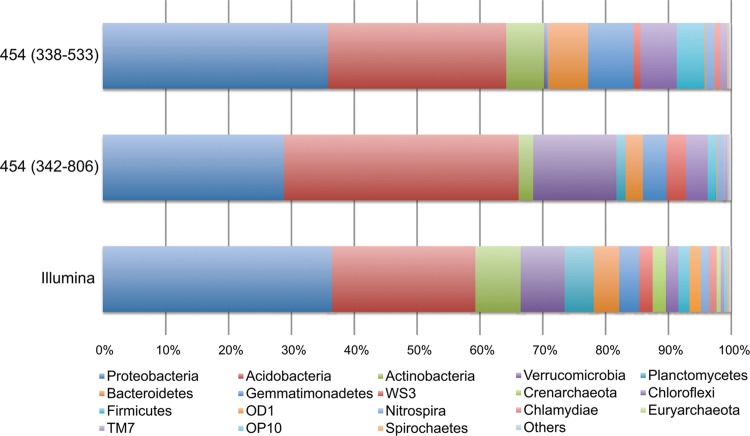
Phylum-level taxonomic compositions of prokaryotes in the soil metagenome on the basis of the 338F-533R and 342F-806R amplicon pyrosequencing and the 16S rRNA gene fragments from Illumina metagenomic sequencing. The top 18 abundant phyla from the results of Illumina metagenomic sequencing are listed.

**Figure 4.**
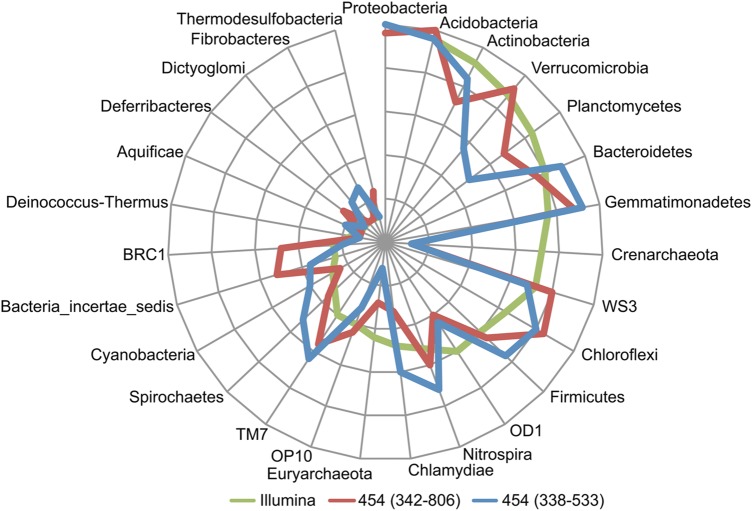
Comparison of three experiments for rank abundances of phyla. Large circles indicate the rank abundances of each phylum in the three experiments, starting at the rank abundance 1, followed by 6, 11, 16, 21, and 26. Phyla are listed in a clockwise direction according to the rank abundance in the Illumina experiment.

Figure 3 shows the results of a BLASTN search for phylum-level taxonomic assignment of each 454 and Illumina read against our merged in-house high-quality RDP and CRW sequence database. The numbers of taxonomically assigned reads at the phylum level were 13 591 (89.62% of the total high-quality 454 reads for the 338F-533R experiment), 26 738(97.13% of the total high-quality 454 reads for the 342F-806R experiment), and 1513 for the Illumina read pairs (3026 reads) (Supplementary Table S6). Among the three types of experiments, the taxonomic compositions at the phylum level exhibited good Spearman's correlations (*P*-value < 0.05): illumina versus 338F-533R, *r* = 0.75; Illumina versus 342F-806R, *r* = 0.81; and 338F-533R versus 342F-806R, *r* = 0.86. These three experiments identified *Proteobacteria* and *Acidobacteria* as the major phyla in the soil microbial community. The compositional bias for each phylum among the three experiments was evaluated by investigating the rank abundance of each phylum in each experiment (Fig. 4). In comparison with the Illumina metagenomic sequencing data, the 454 amplicon sequencing using the 338F-533R and 342F-806R primer sets gave rise to data in which the phyla *Verrucomicrobia*, *Planctomycetes*, *Crenarchaeota* and *Euryarchaeaota*, and the phyla *Planctomycetes*, *Crenarchaeota*, OD1, and *Chlamydiae*, respectively, were underrepresented. Such underrepresentation might have occurred because most 16S rRNA genes from the phyla had sequences with more than two mismatches with the 338F and 342F primer sequences. The use of the 338F-533R primer set generated no 454 reads that were assigned to *Euryarchaeota*, being consistent with the specific amplification of only bacterial 16S rRNA genes by this primer set. Conversely, eight 454 reads by the 342F-806R experiment and ten read pairs by the Illumina metagenomic sequencing were assigned to this phylum, indicating that 342F-806R can simultaneously amplify bacterial and archaeal 16S rRNA genes.

The genus-level taxonomic assignment for the 454 and Illumina reads is depicted in Supplementary Figure S3. The numbers of reads assigned at the genus level were 9956 (65.65% of the total high-quality 454 reads for the 338F-533R experiment), 20 431(74.22% of the total high-quality 454 reads for the 342F-806R experiment), and 1041 (Illumina read pairs) (Supple-mentary Table S7). The top 20 abundant genera identified in each experiment accounted for more than 70% of the taxonomically assigned reads in all three experiments. Most of the top 20 most-abundant genera in each experiment (Supplementary Fig. S4) were also categorized as those in the two other experiments, and a total of the top 26 most-abundant genera were used for comparative analysis. Compared with the Illumina metagenomic sequencing data, the 454 amplicon sequencing data led to an underrepresentation of three genera (the genera *Subdivision3* and *Spartobacteria* in the *Verrucomicrobia* phylum, and the genus *Zavarzinella* in the *Planctomycetes* phylum) and two genera (*Zavarzinella* and OD1 in the OD1 phylum) in the 338F-533R and 342F-806R experiments, respectively. Underrepresentation might have also occurred because the 16S rRNA genes from these genera had sequences with more than two mismatches for the 338F or 342F primer. The 16S rRNA genes in certain genera (e.g. *Conexibacter* and *Zavarzinella*) were underrepresented in the 342F-806R or 338F-533R data when compared with the Illumina metagenomic sequencing data, and their underrepresentation could not be explained by the primer mismatches. The formation of (a) specific secondary structure(s) or differences in the accuracies of the taxonomic assignments for the 454 and Illumina reads might have been responsible for the underrepresentation. The Spearman's correlations calculated for the abundance of the 26 genera between each pair of experiments were: 0.37 (Illumina versus 338F-533R), 0.71 (Illumina versus 342F-806R), and 0.60 (338F-533R versus 342F-806R). Comparison at the genus-levels indicated that the taxonomic composition inferred by the 338F-533R amplicon sequencing correlated weakly with that by the Illumina metagenomic sequencing. In contrast, the taxonomic composition by the 342F-806R amplicon sequencing correlated very well with that by the Illumina metagenomic sequencing, indicating that our universal primers can amplify a broad range of taxa with a low bias.

Since the Illumina sequencing of 16S rRNA gene fragments in the metagenome produced no chimera or bias that is associated with the initial PCR step of amplicon sequencing, the taxonomic composition of the microbial community based on this sequencing method is highly reliable.^41^ However, the inference of taxonomic composition in a microbial community obtained using the metagenomic 16S rRNA gene fragments requires ultra-deep sequencing since such fragments represent only a very small fraction of the metagenome.^42^ Indeed, our use of the Illumina GA IIx platform for metagenome sequencing of the soil microbial community gave rise to only thousands of reads as 16S rRNA gene fragments from >10 million total reads. This observation implies that the quantification of relative abundance in a microbial community will be reliable for major taxa but unsuitable for minor taxa. Furthermore, the metagenomic 16S rRNA gene fragments corresponded randomly to a number of different regions within the 16S rRNA genes, indicating the difficulty in conducting a detailed comparison of metagenomic 16S rRNA gene fragments (e.g. construction of a phylogenetic tree). To characterize the taxonomic composition of a microbial community including its minor taxa at low cost, the amplification of 16S rRNA genes using appropriate universal primers is necessary. The taxonomic composition inferred by the 454 amplicon sequencing with our non-degenerate universal primer set correlated well with that obtained by Illumina metagenomic sequencing, indicating that our universal primers can characterize microbial communities without substantial bias and with low possibility to amplify eukaryotic rRNA genes. Although we did not carry out additional experimental validation of our novel and non-degenerate universal primer set by using other environmental samples rich in eukaryotic and mitochondrial rRNA genes, the results in this study indicate that our primer set is highly expected to be applicable to the analysis of many diverse microbial communities.

## Supplementary Data

Supplementary data are available at www.dnaresearch.oxfordjournals.org.

## Funding

This work was supported by a Grant-in-Aid for Scientific Research on Innovative Area ‘Genome Science’, Ministry of Education, Culture, Sports, Science and Technology in Japan, and a Grant-in-Aid for the Institute for Bioinformatics Research and Development, the Japan Science and Technology Agency (BIRD-JST).

## Supplementary Material

Supplementary Data

## References

[1] Hamady M., Knight R. (2009). Microbial community profiling for human microbiome projects: tools, techniques, and challenges,. Genome Res..

[2] Rappe M.S., Giovannoni S.J. (2003). The uncultured microbial majority. Annu. Rev. Microbiol..

[3] Hamady M., Walker J.J., Harris J.K., Gold N.J., Knight R. (2008). Error-correcting barcoded primers for pyrosequencing hundreds of samples in multiplex. Nat. Methods.

[4] Mori H., Maruyama F., Kurokawa K. (2010). VITCOMIC: visualization tool for taxonomic compositions of microbial communities based on 16S rRNA gene sequences. BMC Bioinformatics.

[5] Turnbaugh P.J., Hamady M., Yatsunenko T. (2009). A core gut microbiome in obese and lean twins. Nature.

[6] Margulies M., Egholm M., Altman W.E. (2005). Genome sequencing in microfabricated high-density picolitre reactors. Nature.

[7] Kuczynski J., Lauber C.L., Walters W.A. (2012). Experimental and analytical tools for studying the human microbiome. Nat. Rev. Genet..

[8] Liu Z., Lozupone C., Hamady M., Bushman F.D., Knight R. (2007). Short pyrosequencing reads suffice for accurate microbial community analysis. Nucleic Acids Res..

[9] Wang Y., Qian P.Y. (2009). Conservative fragments in bacterial 16S rRNA genes and primer design for 16S ribosomal DNA amplicons in metagenomic studies. PLoS ONE.

[10] Baker G.C., Smith J.J., Cowan D.A. (2003). Review and re-analysis of domain-specific 16S primers. J. Microbiol. Methods.

[11] Kim S.W., Suda W., Kim S. (2013). Robustness of gut microbiota of healthy adults in response to probiotic intervention revealed by high-throughput pyrosequencing. DNA Res..

[12] Huse S.M., Dethlefsen L., Huber J.A., Mark W.D., Relman D.A., Sogin M.L. (2008). Exploring microbial diversity and taxonomy using SSU rRNA hypervariable tag sequencing. PLoS Genet..

[13] Auguet J.C., Barberan A., Casamayor E.O. (2010). Global ecological patterns in uncultured *Archaea*. ISME J..

[14] Swan B.K., Ehrhardt C.J., Reifel K.M., Moreno L.I., Valentine D.L. (2010). Archaeal and bacterial communities respond differently to environmental gradients in anoxic sediments of a California hypersaline lake, the Salton Sea. Appl. Environ. Microbiol..

[15] Bates S.T., Berg-Lyons D., Caporaso J.G., Walters W.A., Knight R., Fierer N. (2011). Examining the global distribution of dominant archaeal populations in soil. ISME J..

[16] Walters W.A., Caporaso J.G., Lauber C.L., Berg-Lyons D., Fierer N., Knight R. (2011). PrimerProspector: de novo design and taxonomic analysis of barcoded polymerase chain reaction primers. Bioinformatics.

[17] Klindworth A., Pruesse E., Schweer T. (2012). Evaluation of general 16S ribosomal RNA gene PCR primers for classical and next-generation sequencing-based diversity studies. Nucleic Acids Res..

[18] Huys G., Vanhoutte T., Joossens M. (2008). Coamplification of eukaryotic DNA with 16S rRNA gene-based PCR primers: possible consequences for population fingerprinting of complex microbial communities. Curr. Microbiol..

[19] Prosdocimi E.M., Novati S., Bruno R. (2013). Errors in ribosomal sequence datasets generated using PCR-coupled “panbacterial’ pyrosequencing, and the establishment of an improved approach. Mol. Cell Probes.

[20] Galkiewicz J.P., Kellogg C.A. (2008). Cross-kingdom amplification using bacteria-specific primers: complications for studies of coral microbial ecology. Appl. Environ. Microbiol..

[21] The Human Microbiome Project Consortium (2012). A framework for human microbiome research. Nature.

[22] Kumar P.S., Brooker M.R., Dowd S.E., Camerlengo T. (2011). Target region selection is a critical determinant of community fingerprints generated by 16S pyrosequencing. PLoS ONE.

[23] de Lillo A., Ashley F.P., Palmer R.M. (2006). Novel subgingival bacterial phylotypes detected using multiple universal polymerase chain reaction primer sets. Oral Microbiol. Immunol..

[24] Bennett S. (2004). Solexa Ltd. Pharmacogenomics.

[25] Lagesen K., Hallin P., Rodland E.A., Staerfeldt H.H., Rognes T., Ussery D.W. (2007). RNAmmer: consistent and rapid annotation of ribosomal RNA genes. Nucleic Acids Res..

[26] Yarza P., Richter M., Peplies J. (2008). The All-Species Living Tree project: a 16S rRNA-based phylogenetic tree of all sequenced type strains. Syst. Appl. Microbiol..

[27] Acinas S.G., Marcelino L.A., Klepac-Ceraj V., Polz M.F. (2004). Divergence and redundancy of 16S rRNA sequences in genomes with multiple *rrn* operons. J. Bacteriol..

[28] Katoh K., Toh H. (2008). Improved accuracy of multiple ncRNA alignment by incorporating structural information into a MAFFT-based framework. BMC Bioinformatics.

[29] Cole J.R., Wang Q., Cardenas E. (2009). The Ribosomal Database Project: improved alignments and new tools for rRNA analysis. Nucleic Acids Res..

[30] Cannone J.J., Subramanian S., Schnare M.N. (2002). The comparative RNA web (CRW) site: an online database of comparative sequence and structure information for ribosomal, intron, and other RNAs. BMC Bioinformatics.

[31] Claesson M.J., Wang Q., O'Sullivan O. (2010). Comparison of two next-generation sequencing technologies for resolving highly complex microbiota composition using tandem variable 16S rRNA gene regions. Nucleic Acids Res..

[32] Wang Y., Shimodaira J., Miyasaka T. (2008). Detection of *bphAa* gene expression of *Rhodococcus* sp. strain RHA1 in soil using a new method of RNA preparation from soil. Biosci. Biotechnol. Biochem..

[33] Schloss P.D., Westcott S.L., Ryabin T. (2009). Introducing mothur: open-source, platform-independent, community-supported software for describing and comparing microbial communities. Appl. Environ. Microbiol..

[34] Altschul S.F., Gish W., Miller W., Myers E.W., Lipman D.J. (1990). Basic local alignment search tool. J. Mol. Biol..

[35] Itoh T., Suzuki K., Nakase T. (1998). Occurrence of introns in the 16S rRNA genes of members of the genus *Thermoproteus*. Arch. Microbiol..

[36] Mao D.P., Zhou Q., Chen C.Y., Quan Z.X. (2012). Coverage evaluation of universal bacterial primers using the metagenomic datasets. BMC Microbiol..

[37] Frank J.A., Reich C.I., Sharma S., Weisbaum J.S., Wilson B.A., Olsen G.J. (2008). Critical evaluation of two primers commonly used for amplification of bacterial 16S rRNA genes. Appl. Environ. Microbiol..

[38] Schneider T.D., Stephens R.M. (1990). Sequence logos: a new way to display consensus sequences. Nucleic Acids Res..

[39] King G.M., Judd C., Kuske C.R., Smith C. (2012). Analysis of stomach and gut microbiomes of the eastern oyster (*Crassostrea virginica*) from coastal Louisiana. PLoS ONE.

[40] Gribaldo S., Brochier-Armanet C. (2006). The origin and evolution of *Archaea*: a state of the art. Philos. Trans. R. Soc. Lond. B Biol. Sci..

[41] Hamp T.J., Jones W.J., Fodor A.A. (2009). Effects of experimental choices and analysis noise on surveys of the “rare biosphere”. Appl. Environ. Microbiol..

[42] Yooseph S., Nealson K.H., Rusch D.B. (2010). Genomic and functional adaptation in surface ocean planktonic prokaryotes. Nature.

