# Supplementary material for: Design and Experimental Application of a Novel Non-Degenerate Universal Primer Set that Amplifies Prokaryotic 16S rRNA Genes with a Low Possibility to Amplify Eukaryotic rRNA Genes
Source: DNA Res. 2013 Nov 25;21(2):217–27. doi: 10.1093/dnares/dst052 (PMC3989492; doi:10.1093/dnares/dst052)
Supplement: Supplementary Data [file supp_21_2_217__index.html]

Design and Experimental Application of a Novel Non-Degenerate Universal Primer Set that Amplifies Prokaryotic 16S rRNA Genes with a Low Possibility to Amplify Eukaryotic rRNA Genes — Design and Experimental Application of a Novel Non-Degenerate Universal Primer Set that Amplifies Prokaryotic 16S rRNA Genes with a Low Possibility to Amplify Eukaryotic rRNA Genes — Supplementary Data 

# Design and Experimental Application of a Novel Non-Degenerate Universal Primer Set that Amplifies Prokaryotic 16S rRNA Genes with a Low Possibility to Amplify Eukaryotic rRNA Genes

## Supplementary Data

Supplementary Data

**Files in this Data Supplement:**

- Supplementary Figure 1 - pdf file
- Supplementary Figure 2 - pdf file
- Supplementary Figure 3 - pdf file
- Supplementary Figure 4 - pdf file
- Supplementary Data - Supplementary Data
- Supplementary Table 1 - doc file
- Supplementary Table 2 - doc file
- Supplementary Table 3 - xls file
- Supplementary Table 4 - xls file
- Supplementary Table 5 - xls file
- Supplementary Table 6 - doc file
- Supplementary Table 7 - doc file
