# Supplementary material for: Design and Experimental Application of a Novel Non-Degenerate Universal Primer Set that Amplifies Prokaryotic 16S rRNA Genes with a Low Possibility to Amplify Eukaryotic rRNA Genes
Source: DNA Res. 2013 Nov 25;21(2):217–27. doi: 10.1093/dnares/dst052 (PMC3989492; doi:10.1093/dnares/dst052)
Supplement: Supplementary Data [file supp_dst052_dst052supp_fig1.pdf]

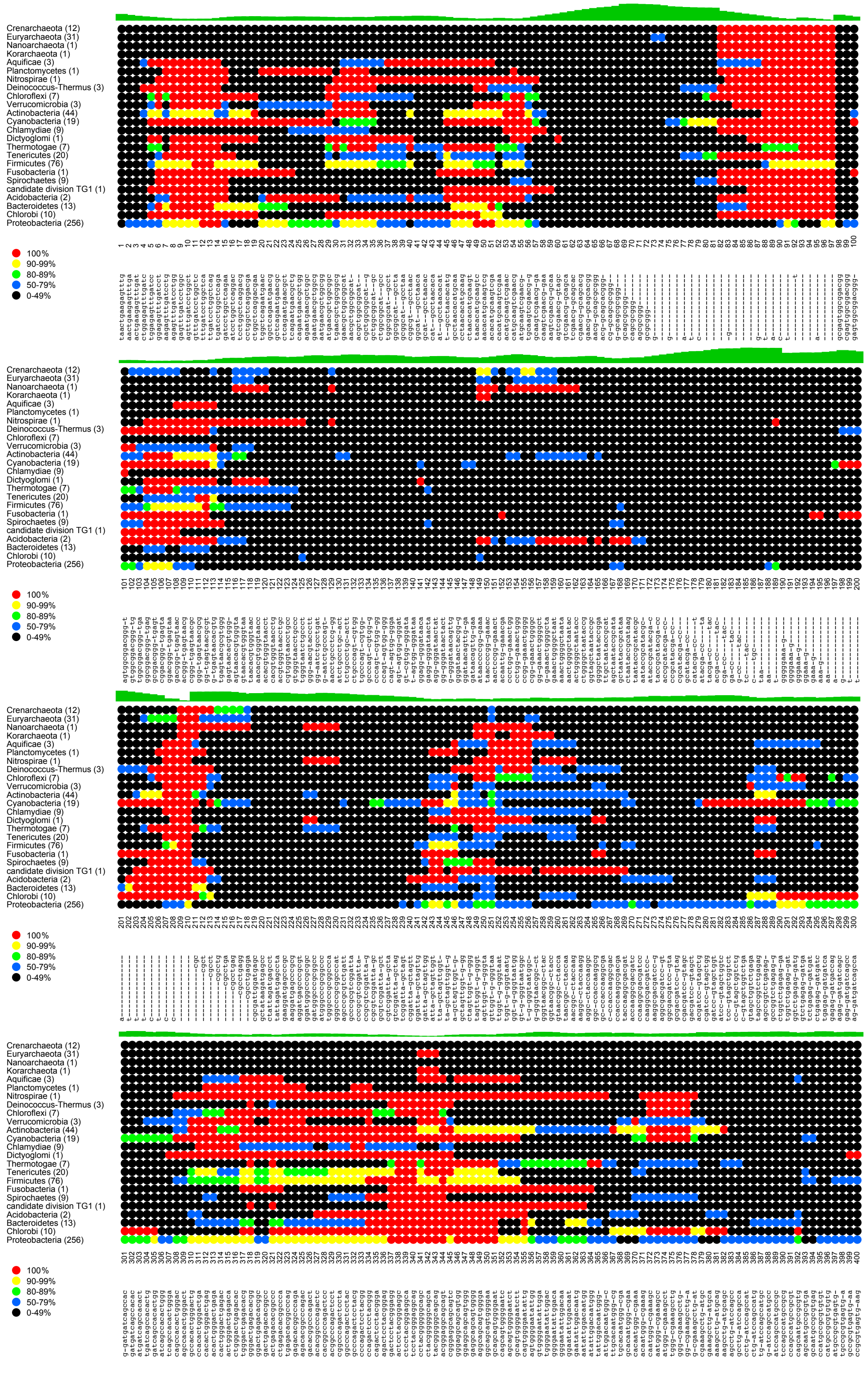


**Fig. S1.** A complete plot of the consensus sequence in each window and the coverage rates for each phylum from the 531 16S rRNA genes of genome-sequenced strains. Some consensus sequences contain continuous gaps (-) with several bp in lengths, indicating the presence of inserted sequences in particular strains. See the legend to Fig. 1 for the dot colors and the bar graph.

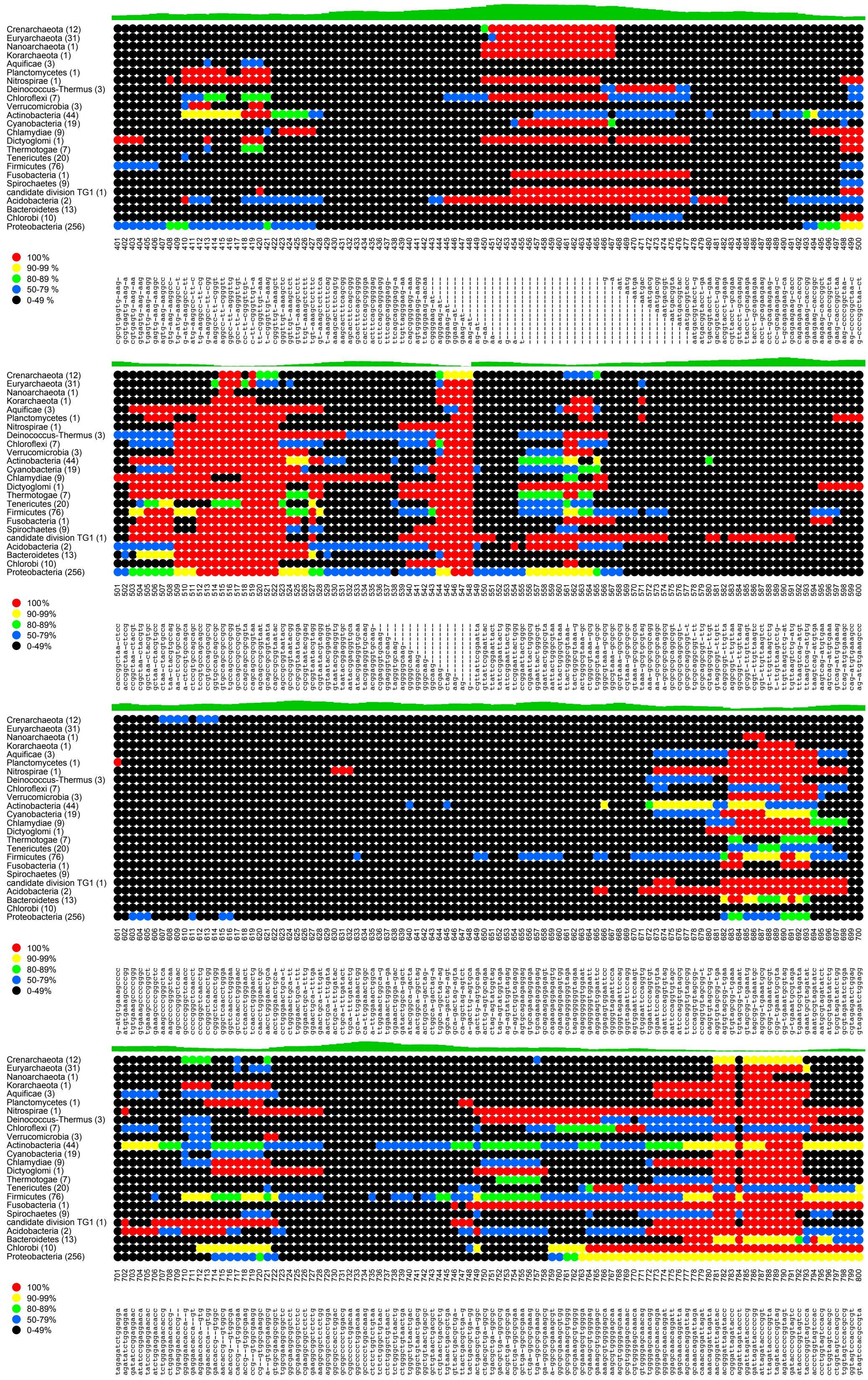


**Fig. S1.** Continued.

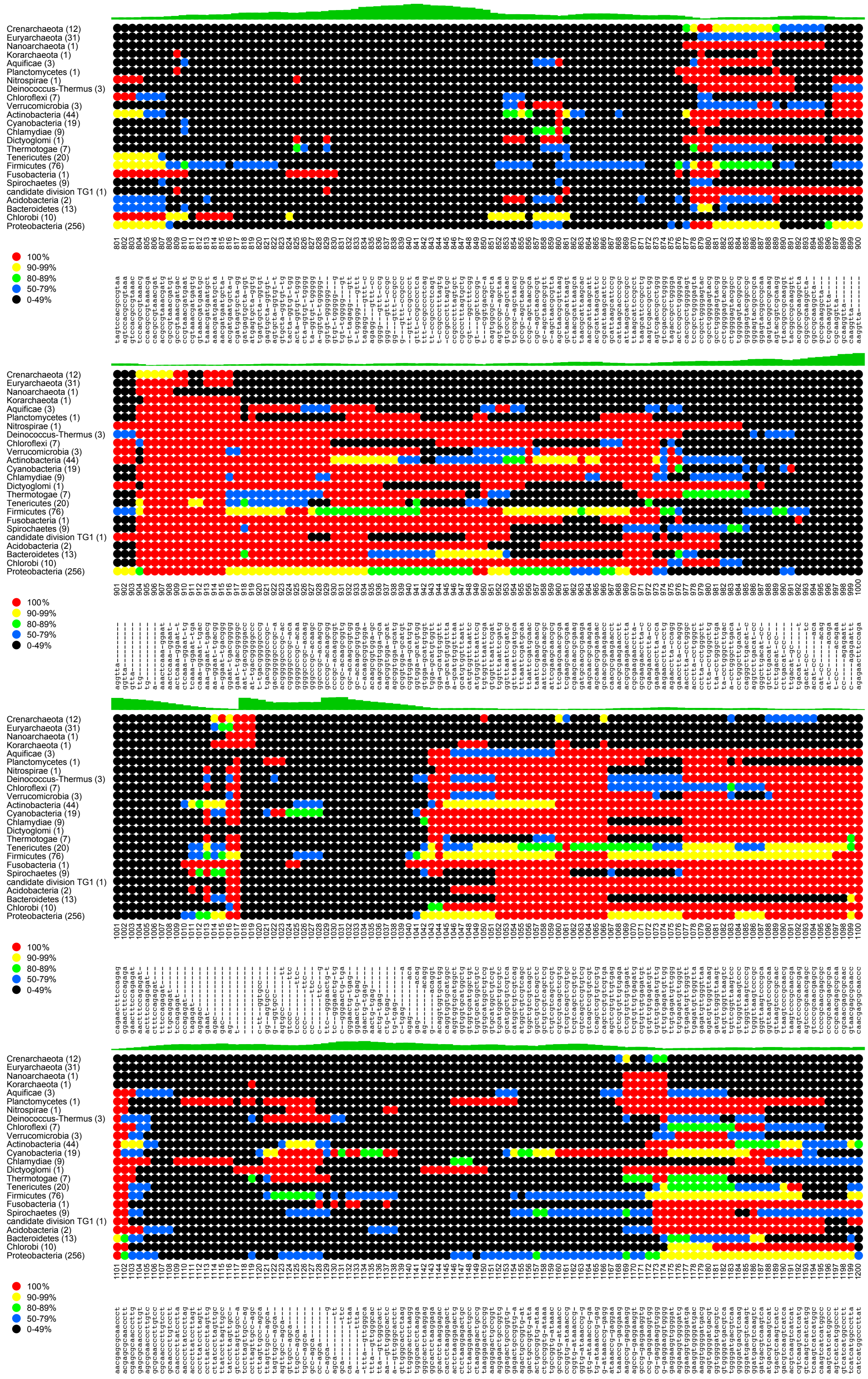


**Fig. S1.** Continued.

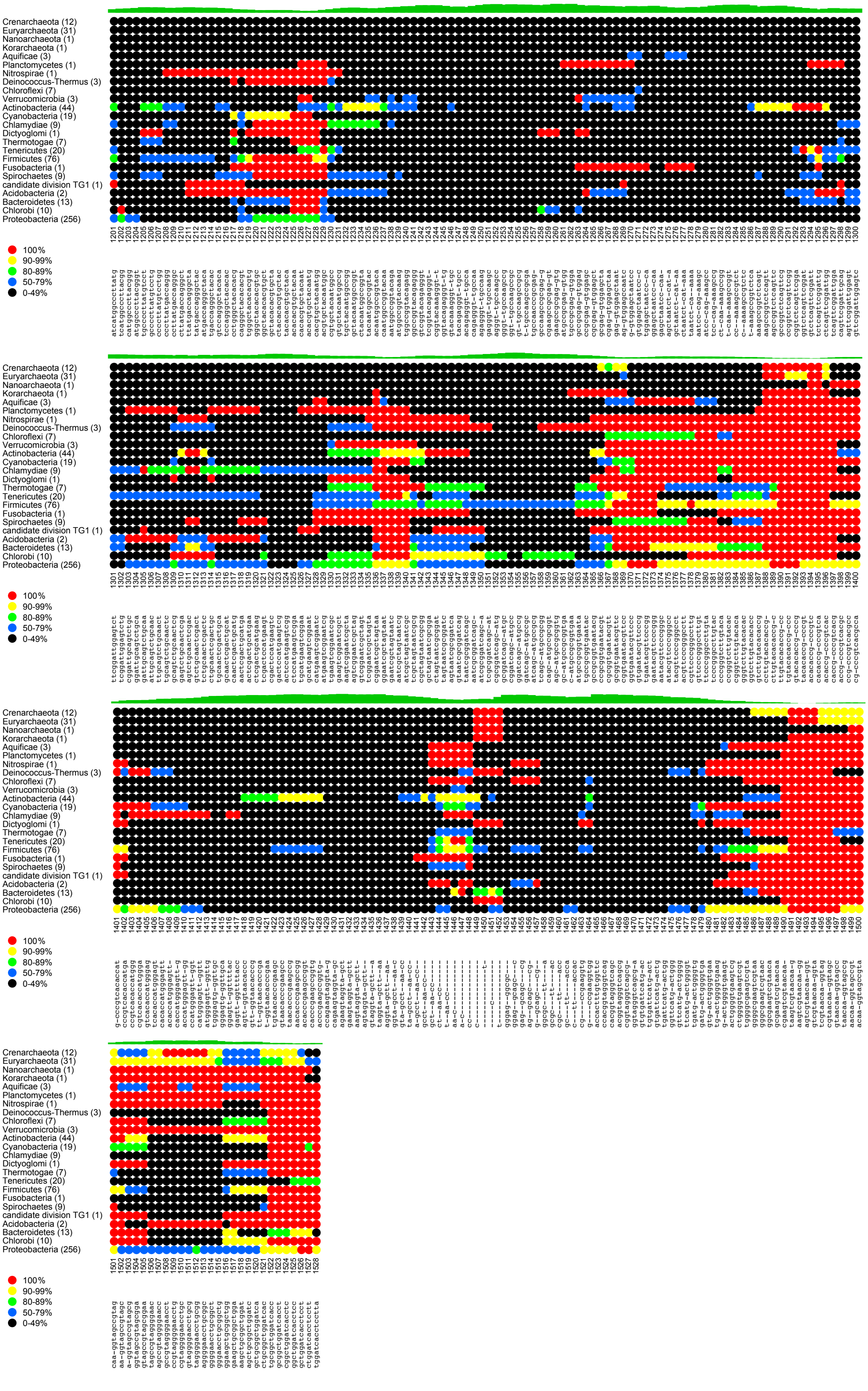


**Fig. S1.** Continued.
