# Supplementary material for: Design and Experimental Application of a Novel Non-Degenerate Universal Primer Set that Amplifies Prokaryotic 16S rRNA Genes with a Low Possibility to Amplify Eukaryotic rRNA Genes
Source: DNA Res. 2013 Nov 25;21(2):217–27. doi: 10.1093/dnares/dst052 (PMC3989492; doi:10.1093/dnares/dst052)
Supplement: Supplementary Data [file supp_dst052_dst052supp_fig2.pdf]

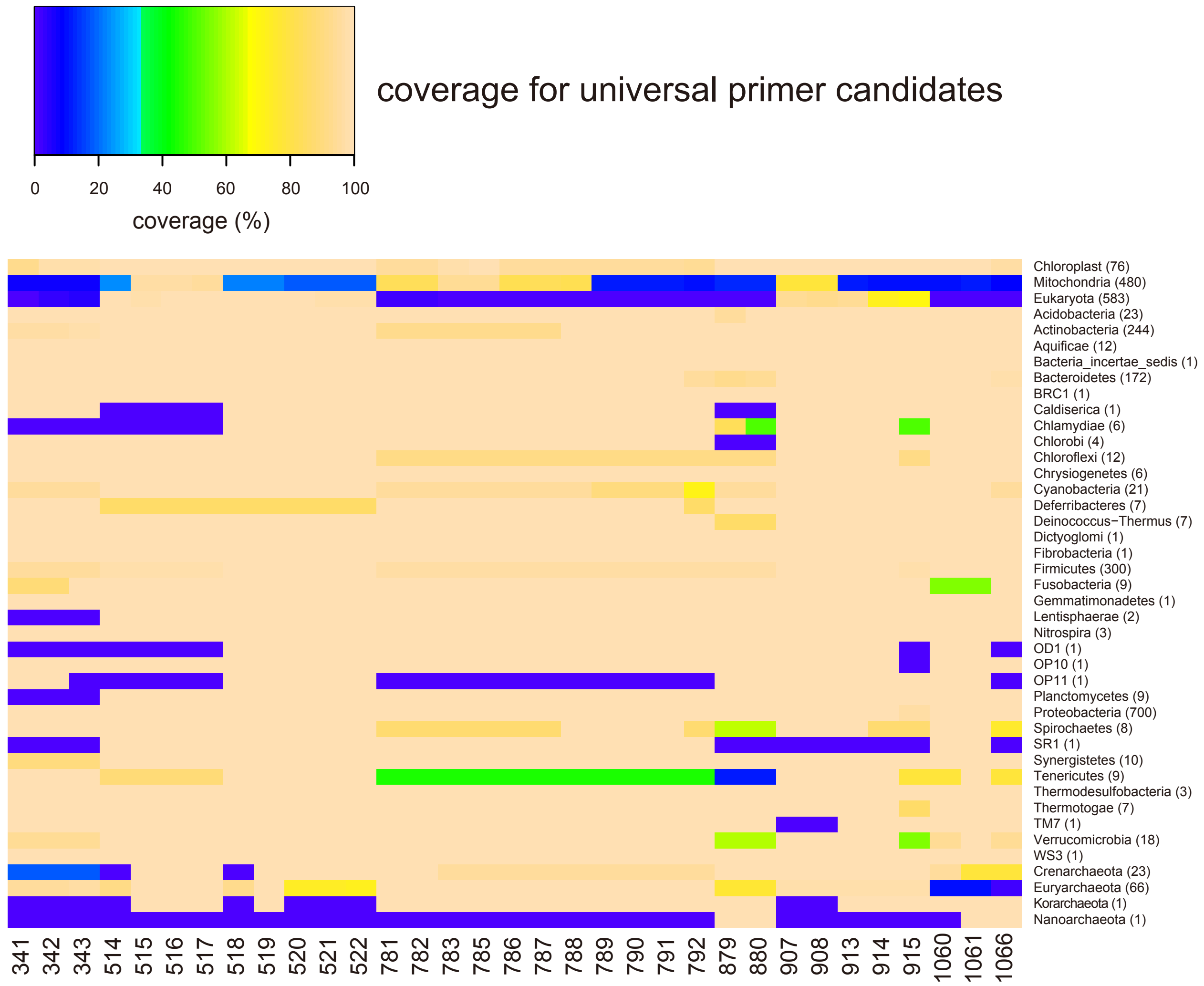

Fig. S2. The coverage for the 33 candidate sequences of universal primers in each of the bacterial and archaeal phyla rRNA genes in our in-house high quality RDP database, and the chloroplastic, mitochondrial, and eukaryotic rRNA genes in the CRW-derived and above-descrived database. Each column indicates the coverage for a candidate sequence from each of the bacterial and archaeal phyla rRNA genes, and the chloroplastic, mitochondrial, and eukaryotic rRNA genes by color intensity: blue = low coverage, and pale orange = high coverage. The number of genera is provided in parentheses.
