# Supplementary material for: Design and Experimental Application of a Novel Non-Degenerate Universal Primer Set that Amplifies Prokaryotic 16S rRNA Genes with a Low Possibility to Amplify Eukaryotic rRNA Genes
Source: DNA Res. 2013 Nov 25;21(2):217–27. doi: 10.1093/dnares/dst052 (PMC3989492; doi:10.1093/dnares/dst052)
Supplement: Supplementary Data [file supp_dst052_dst052supp_fig3.pdf]

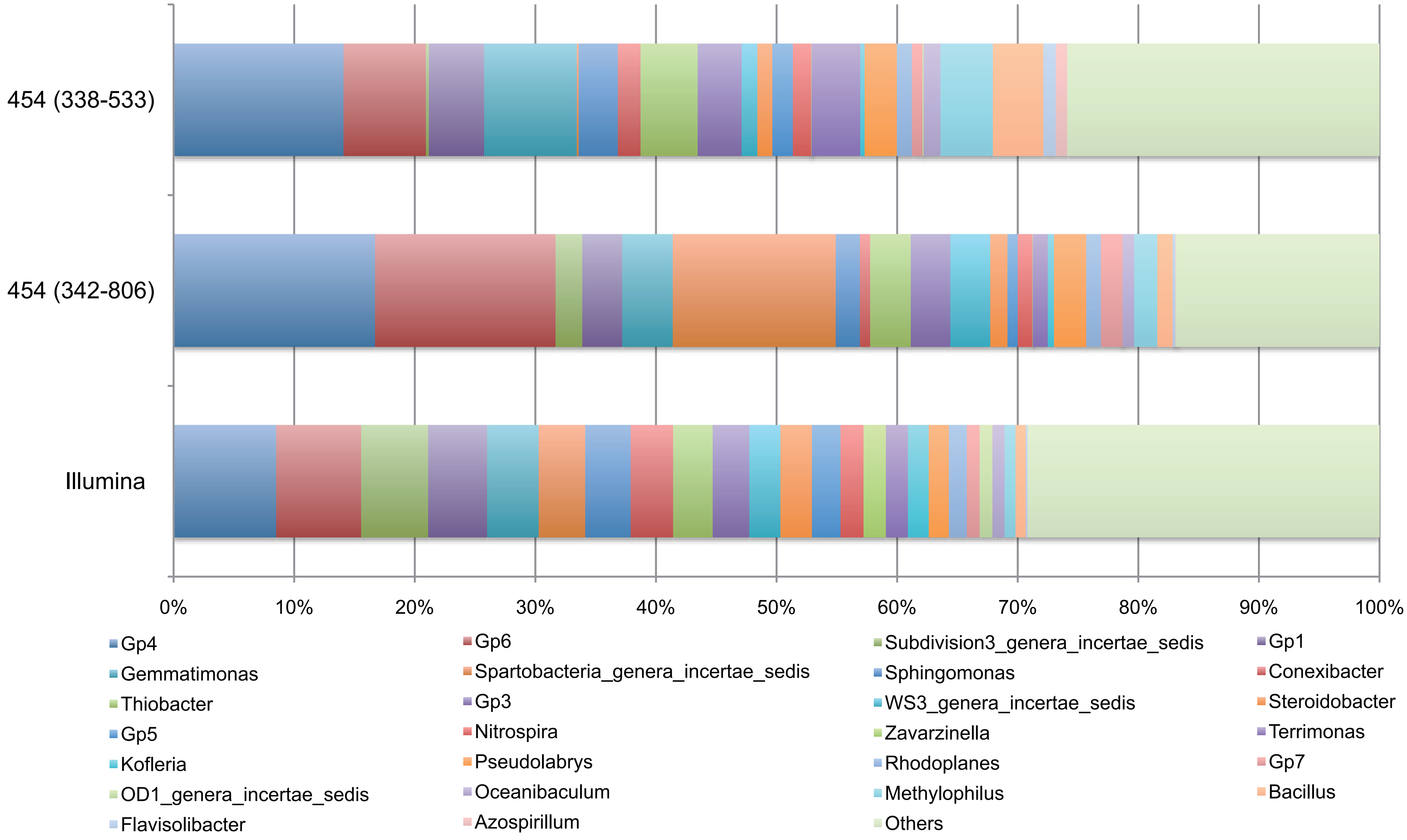

Fig. S3. 16S rRNA-based genus-level taxonomic compositions by the 338F-533R and 342F-806R amplicon sequencing and by the Illumina metagenomic sequencing. The top 26 genera that were found among the top 20 most-abundant genera in the three experiments are represented.
