# Supplementary material for: Design and Experimental Application of a Novel Non-Degenerate Universal Primer Set that Amplifies Prokaryotic 16S rRNA Genes with a Low Possibility to Amplify Eukaryotic rRNA Genes
Source: DNA Res. 2013 Nov 25;21(2):217–27. doi: 10.1093/dnares/dst052 (PMC3989492; doi:10.1093/dnares/dst052)
Supplement: Supplementary Data [file supp_dst052_dst052supp_fig4.pdf]

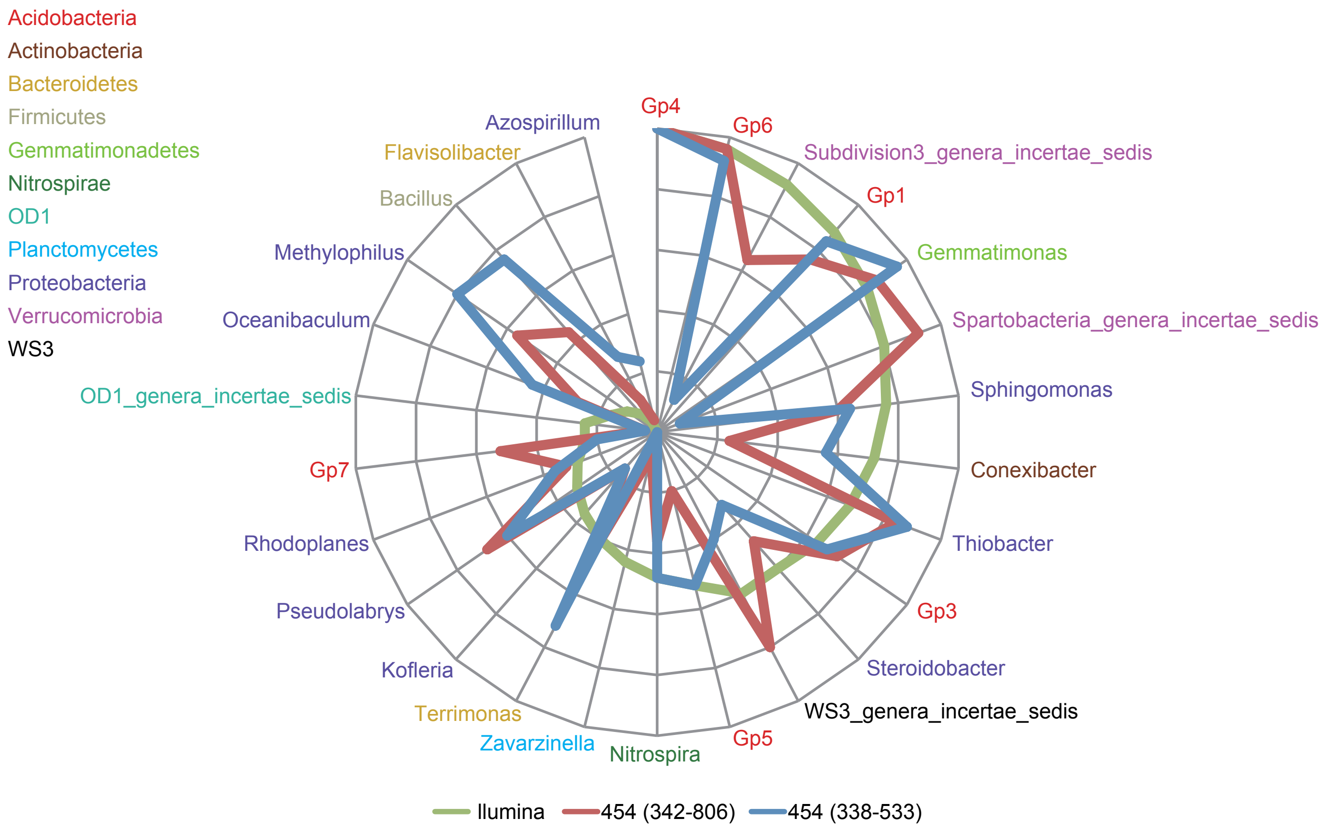

Fig. S4. Comparison of the three sets of experimental results for rank abundance in the 26 genera that were found among the top 20 most-abundant genera in the three experiments. Large circles indicate the rank abundances of each phylum in one of the three experiments, starting at the rank abundance 1, followed by 6, 11, 16, 21, and 26. The font color for each genus name corresponds to its phylum name.
