# Supplementary material for: Design and Experimental Application of a Novel Non-Degenerate Universal Primer Set that Amplifies Prokaryotic 16S rRNA Genes with a Low Possibility to Amplify Eukaryotic rRNA Genes
Source: DNA Res. 2013 Nov 25;21(2):217–27. doi: 10.1093/dnares/dst052 (PMC3989492; doi:10.1093/dnares/dst052)
Supplement: Supplementary Data [file supp_dst052_dst052supp_table1.doc]

**Table S1.** The sequences of 19 universal primers.

| Primer | Sequence (5' to 3') | Original sequence *^a^* | Length | Position | Reference |
| --- | --- | --- | --- | --- | --- |
| 338F | ACTCCTACGGGAGGCAGCAG | The same | 20 | 338-357 | 3 |
| 341F | CCTACGGGGGGCAGCAG | CCTACGGGRSGCAGCAG | 17 | 341-357 | 4 |
| 342F | CTACGGGGGGCAGCAG |  | 16 | 342-357 | This study |
| 357R | CTGCTGCCTCCCGTA | CTGCTGCCTYCCGTA | 15 | 343-357 | 2 |
| 519F | CAGCAGCCGCGGTAATAC | CAGCMGCCGCGGTAATWC | 18 | 519-536 | 4 |
| 529R | ACCGCGGCGGCTGGC | ACCGCGGCKGCTGGC | 15 | 517-531 | 1 |
| 533R | TTACCGCGGCTGCTGGCAC | The same | 19 | 515-533 | 3 |
| 534R | ATTACCGCGGCTGCTGG | The same | 17 | 518-534 | 2 |
| 563F | ACTGGGCGTAAAGGG | AYTGGGYDTAAAGNG | 15 | 563-577 | 2 |
| 779F | GCAAACCGGATTAGATACCC | GCRAASSGGATTAGATACCC | 20 | 778-797 | 1 |
| 784F | AGGATTAGATACCCC | RGGATTAGATACCC | 15 | 784-798 | 2 |
| 789F | TAGATACCCCGGTAGTCC | TAGATACCCSSGTAGTCC | 18 | 789-806 | 4 |
| 802R | TACCAGGGTATCTAATCC | TACNVGGGTATCTAATCC | 18 | 785-802 | 2 |
| 806R | GGACTACCGGGGTATCT |  | 17 | 790-806 | This study |
| 906F | GAAACTTAAAGGAATTG | GAAACTTAAAKGAATTG | 17 | 906-922 | 4 |
| 926R | CCGTCAATTCCTTTAAGTTT | CCGTCAATTYYTTTRAGTTT | 20 | 907-926 | 2 |
| 1053F | GCATGGCTGTCGTCAG | GCATGGCYGYCGTCAG | 16 | 1053-1068 | 4 |
| 1064R | CGACAGCCATGCACCACCT | CGACRRCCATGCANCACCT | 19 | 1046-1064 | 2 |
| 1099F | GTAACGAGCGCAACCC | GYAACGAGCGCAACCC | 16 | 1099-1114 | 2 |

*^a^* The original degenerate sequence of the primer in the reference.

**References**

1. Baker, G.C., Smith, J.J., and Cowan, D.A.. 2003, Review and re-analysis of domain-specific 16S primers. *J. Microbiol. Methods,* **55**, 541–555.

2. Claesson, M.J., Wang, Q., O'Sullivan, O., Greene-Diniz, R., Cole, J.R., Ross, R.P., and O'Toole P.W.. 2010, Comparison of two next-generation sequencing technologies for resolving highly complex microbiota composition using tandem variable 16S rRNA gene regions. *Nucleic Acids Res.*, **38**, e200.

3. Huse, S.M., Dethlefsen, L., Huber, J.A., Mark, W.D., Relman, D.A., and Sogin, M.L. 2008, Exploring microbial diversity and taxonomy using SSU rRNA hypervariable tag sequencing. *PLoS Genet.*, **4**, e1000255.

4. Wang, Y., and Qian, P.Y. 2009, Conservative fragments in bacterial 16S rRNA genes and primer design for 16S ribosomal DNA amplicons in metagenomic studies. *PLoS One*, **4**, e7401.
