# Supplementary material for: Design and Experimental Application of a Novel Non-Degenerate Universal Primer Set that Amplifies Prokaryotic 16S rRNA Genes with a Low Possibility to Amplify Eukaryotic rRNA Genes
Source: DNA Res. 2013 Nov 25;21(2):217–27. doi: 10.1093/dnares/dst052 (PMC3989492; doi:10.1093/dnares/dst052)
Supplement: Supplementary Data [file supp_dst052_dst052supp_table2.doc]

**Table S2.** The 50 candidate sequences of universal primers.

| Start Position | Sequence (5' to 3') |
| --- | --- |
| 341 | CCTACGGGGGGCAGC |
| 342 | CTACGGGGGGCAGCA |
| 343 | TACGGGGGGCAGCAG |
| 514 | GGTGCCAGCAGCCGC |
| 515 | GTGCCAGCCGCCGCG |
| 516 | TGCCAGCCGCCGCGG |
| 517 | GCCAGCCGCCGCGGT |
| 518 | CCAGCAGCCGCGGTA |
| 519 | CAGCAGCCGCGGTAA |
| 520 | AGCAGCCGCGGTAAT |
| 521 | GCAGCCGCGGTAATA |
| 522 | CAGCCGCGGTAATAC |
| 781 | AACGGGATTAGATAC |
| 782 | ACGGGATTAGATACC |
| 783 | CGGGATTAGATACCC |
| 785 | GGATTAGATACCCCG |
| 786 | GATTAGATACCCCGG |
| 787 | ATTAGATACCCCGGT |
| 788 | TTAGATACCCCGGTA |
| 789 | TAGATACCCCGGTAG |
| 790 | AGATACCCCGGTAGT |
| 791 | GATACCCCGGTAGTC |
| 792 | ATACCCCGGTAGTCC |
| 879 | CCGCCTGGGGAGTAC |
| 880 | CGCCTGGGGAGTACG |
| 907 | AAACTCAAAGGAATT |
| 908 | AACTCAAAGGAATTG |
| 913 | AAAGGAATTGACGGG |
| 914 | AAGGAATTGACGGGG |
| 915 | AGGAATTGACGGGGG |
| 1060 | CGTCGTCAGCTCGTG |
| 1061 | GTCGTCAGCTCGTGC |
| 1066 | CAGCTCGTGTCGTGA |
| 1388 | CCTTGTACACACCGC |
| 1389 | CTTGTACACACCGCC |
| 1390 | TTGTACACACCGCCC |
| 1391 | TGTACACACCGCCCG |
| 1392 | GTACACACCGCCCGT |
| 1393 | TACACACCGCCCGTC |
| 1394 | ACACACCGCCCGTCA |
| 1395 | CACACCGCCCGTCAC |
| 1491 | TAAGTCGTAACAAGG |
| 1492 | AAGTCGTAACAAGGT |
| 1493 | AGTCGTAACAAGGTA |
| 1494 | GTCGTAACAAGGTAG |
| 1495 | TCGTAACAAGGTAGC |
| 1496 | CGTAACAAGGTAGCC |
| 1525 | GGCTGGATCACCTCC |
| 1526 | GCTGGATCACCTCCT |
| 1527 | CTGGATCACCTCCTT |
