# Supplementary material for: Design and Experimental Application of a Novel Non-Degenerate Universal Primer Set that Amplifies Prokaryotic 16S rRNA Genes with a Low Possibility to Amplify Eukaryotic rRNA Genes
Source: DNA Res. 2013 Nov 25;21(2):217–27. doi: 10.1093/dnares/dst052 (PMC3989492; doi:10.1093/dnares/dst052)
Supplement: Supplementary Data [file supp_dst052_dst052supp_table6.doc]

**Table S6.** Phylum-level taxonomic compositions of prokaryotes in the soil metagenome by the 338F-533R and 342F-806R amplicon pyrosequencing and the 16S rRNA gene fragments from Illumina metagenomic sequencing.

| Phylum name*^a^* | 454 (342F-806R) | 454 (338F-533R)*^b^* | Illumina |
| --- | --- | --- | --- |
| *Proteobacteria* | 7,711 | 4868.33 | 551.83 |
| *Acidobacteria* | 9,987 | 3858.33 | 345.33 |
| *Actinobacteria* | 618 | 814.96 | 109 |
| *Verrucomicrobia* | 3,538 | 65 | 106 |
| *Planctomycetes* | 394 | 28 | 70 |
| *Bacteroidetes* | 736 | 863 | 61.33 |
| *Gemmatimonadetes* | 1,008 | 981 | 48.5 |
| WS3 | 816 | 150 | 32 |
| *Crenarchaeota* | 0 | 0 | 32 |
| *Chloroflexi* | 935 | 786.30 | 30 |
| *Firmicutes* | 369 | 602.94 | 27 |
| OD1 | 36 | 25 | 27 |
| *Nitrospira* | 277 | 185.5 | 20 |
| *Chlamydiae* | 11 | 132 | 18 |
| *Euryarchaeota* | 8 | 0 | 10 |
| TM7 | 136 | 141.5 | 8 |
| OP10 | 38 | 7.13 | 8 |
| *Spirochaetes* | 31 | 33 | 4.5 |
| *Cyanobacteria* | 1 | 20 | 2 |
| *Bacteria_incertae_sedis* | 47 | 14 | 1 |
| BRC1 | 39 | 2 | 1 |
| *Deinococcus-Thermus* | 0 | 0 | 0.5 |
| *Deferribacteres* | 1 | 0 | 0 |
| *Dictyoglomi* | 0 | 4 | 0 |
| *Aquificae* | 0 | 2 | 0 |
| *Fibrobacteres* | 0 | 7 | 0 |
| *Thermodesulfobacteria* | 1 | 0 | 0 |
| Total | 26,738 | 13,591 | 1,513 |

*^a^* Phyla in the table are represented in descending order of abundance in the Illumina experiment.

*^b^* Each value is rounded off to two decimal places.
