# Supplementary material for: Design and Experimental Application of a Novel Non-Degenerate Universal Primer Set that Amplifies Prokaryotic 16S rRNA Genes with a Low Possibility to Amplify Eukaryotic rRNA Genes
Source: DNA Res. 2013 Nov 25;21(2):217–27. doi: 10.1093/dnares/dst052 (PMC3989492; doi:10.1093/dnares/dst052)
Supplement: Supplementary Data [file supp_dst052_dst052supp_table7.doc]

**Table S7.** Genus-level taxonomic compositions of prokaryotes in the soil metagenome by the 338F-533R and 342F-806R amplicon pyrosequencing and the 16S rRNA gene fragments from Illumina metagenomic sequencing.

| Genus name | 454 (342F-806R)*^a^* | 454 (338F-533R) | Illumina | Rank abundance in the 454 (342F-806R) experiment*^b^* | Rank abundance in the 454 (338F-533R) experiment | | Rank abundance in the Illumina experiment | |
| --- | --- | --- | --- | --- | --- | --- | --- | --- |
| Gp4 | 3,415 | 1,405 | 88.67 | 1 | | 1 | | 1 |
| Gp6 | 3,062 | 681 | 73.33 | 2 | | 3 | | 2 |
| Subdivision3_genera_incertae_sedis | 451 | 24 | 58 | 10 | | 49 | | 3 |
| Gp1 | 673 | 456 | 50.83 | 7 | | 5 | | 4 |
| *Gemmatimonas* | 860 | 763 | 44.5 | 4 | | 2 | | 5 |
| *Spartobacteria_genera_incertae_sedis* | 2760 | 16 | 40 | 3 | | 63 | | 6 |
| *Sphingomonas* | 407.25 | 324.35 | 39.33 | 11 | | 10 | | 7 |
| *Conexibacter* | 176 | 186.08 | 36.83 | 20 | | 12 | | 8 |
| *Thiobacter* | 688 | 474 | 34 | 5 | | 4 | | 9 |
| Gp3 | 671 | 360 | 31.5 | 8 | | 9 | | 10 |
| *Steroidobacter* | 288 | 123 | 27 | 14 | | 18 | | 11 |
| WS3_genera_incertae_sedis | 678 | 132 | 27 | 6 | | 16 | | 11 |
| Gp5 | 175 | 171 | 24.67 | 21 | | 13 | | 12 |
| *Nitrospira* | 251 | 150 | 20 | 17 | | 14 | | 13 |
| *Zavarzinella* | 5 | 2.33 | 19.5 | 87 | | 131 | | 14 |
| *Terrimonas* | 253.97 | 402.28 | 18.84 | 16 | | 8 | | 15 |
| *Kofleria* | 104.5 | 36 | 18 | 29 | | 40 | | 16 |
| *Pseudolabrys* | 544.16 | 266.09 | 17.33 | 9 | | 11 | | 17 |
| *Rhodoplanes* | 248.04 | 123.47 | 15.67 | 18 | | 17 | | 18 |
| Gp7 | 368 | 88 | 11 | 13 | | 22 | | 19 |
| OD1_genera_incertae_sedis | 2 | 10 | 11 | 100 | | 81 | | 19 |
| *Oceanibaculum* | 193.33 | 140.04 | 10.5 | 19 | | 15 | | 20 |
| *Pirellula* | 5 | 5 | 10 | 87 | | 105 | | 21 |
| *Methylophilus* | 395.51 | 431.81 | 9.37 | 12 | | 6 | | 22 |
| *Bacillus* | 260 | 416.25 | 9 | 15 | | 7 | | 23 |
| *Haliangium* | 138.5 | 45 | 9 | 25 | | 35 | | 23 |
| *Parachlamydia* | 0 | 4 | 9 | 116 | | 115 | | 23 |
| *Methylovorus* | 46 | 32 | 8.37 | 43 | | 41 | | 24 |
| *Bellilinea* | 0 | 3 | 8 | 116 | | 125 | | 25 |
| Gp17 | 139 | 24 | 8 | 24 | | 49 | | 25 |
| TM7_genera_incertae_sedis | 76 | 76 | 8 | 34 | | 26 | | 25 |
| *Methylotenera* | 15.88 | 21.86 | 7.62 | 69 | | 53 | | 26 |
| *Stella* | 112.33 | 77.77 | 7.5 | 28 | | 25 | | 27 |
| *Anaeromyxobacter* | 96 | 51 | 7 | 31 | | 33 | | 28 |
| Gp2 | 104 | 56 | 7 | 30 | | 31 | | 28 |
| *Methylibium* | 64 | 41 | 6.5 | 37 | | 36 | | 29 |
| *Aquicella* | 42 | 9 | 6 | 47 | | 84 | | 30 |
| *Byssovorax* | 21.67 | 31.23 | 6 | 62 | | 42 | | 30 |
| Gp22 | 44 | 80 | 6 | 45 | | 24 | | 30 |
| OP10_genera_incertae_sedis | 30 | 3 | 6 | 51 | | 125 | | 30 |
| *Thermofilum* | 0 | 0 | 6 | 116 | | 225 | | 30 |
| *Solirubrobacter* | 60 | 6.92 | 5.83 | 39 | | 93 | | 31 |
| *Hyphomicrobium* | 169 | 60.39 | 5.5 | 22 | | 30 | | 32 |
| Gp10 | 27 | 6 | 5 | 53 | | 97 | | 33 |
| *Iamia* | 41.5 | 87.5 | 5 | 48 | | 23 | | 33 |
| *Natronococcus* | 0 | 0 | 5 | 116 | | 225 | | 33 |
| *Planctomyces* | 3 | 3 | 5 | 95 | | 125 | | 33 |
| *Azospira* | 23.87 | 1.05 | 4.67 | 57 | | 152 | | 34 |
| *Pedomicrobium* | 78 | 30.16 | 4.5 | 33 | | 44 | | 35 |
| *Ferruginibacter* | 30 | 37.74 | 4.23 | 51 | | 39 | | 36 |
| *Bradyrhizobium* | 163.54 | 63.37 | 4 | 23 | | 27 | | 37 |
| *Desulfoglaeba* | 137 | 29 | 4 | 26 | | 46 | | 37 |
| Gp13 | 24 | 25 | 4 | 56 | | 48 | | 37 |
| *Inquilinus* | 60.33 | 40.33 | 4 | 38 | | 37 | | 37 |
| *Caldilinea* | 12 | 29 | 3 | 75 | | 46 | | 38 |
| Gp18 | 3 | 3 | 3 | 95 | | 125 | | 38 |
| *Mycobacterium* | 21 | 62.96 | 3 | 63 | | 28 | | 38 |
| *Nocardioides* | 6 | 20 | 3 | 84 | | 56 | | 38 |
| *Phaselicystis* | 54.5 | 5.48 | 3 | 42 | | 101 | | 38 |
| *Prosthecomicrobium* | 119 | 88.04 | 3 | 27 | | 21 | | 38 |
| *Spirochaeta* | 20 | 15 | 3 | 64 | | 64 | | 38 |
| *Nitrosospira* | 93.08 | 25 | 2.92 | 32 | | 48 | | 39 |
| *Thiobacillus* | 1 | 10 | 2.75 | 103 | | 81 | | 40 |
| *Gemmata* | 0 | 7.67 | 2.5 | 116 | | 89 | | 41 |
| *Rudaea* | 42 | 22 | 2.5 | 47 | | 51 | | 41 |
| *Sphingopyxis* | 0.5 | 7.68 | 2.5 | 108 | | 88 | | 41 |
| *Variovorax* | 13.56 | 10.5 | 2.5 | 72 | | 79 | | 41 |
| *Burkholderia* | 31.32 | 12 | 2 | 50 | | 72 | | 42 |
| *Geobacter* | 22.17 | 6 | 2 | 60 | | 97 | | 42 |
| *Geothrix* | 9 | 5 | 2 | 78 | | 105 | | 42 |
| *Haliscomenobacter* | 59 | 36 | 2 | 40 | | 40 | | 42 |
| *Ilumatobacter* | 24 | 28 | 2 | 56 | | 47 | | 42 |
| *Marmoricola* | 4 | 7.14 | 2 | 92 | | 91 | | 42 |
| *Opitutus* | 2 | 2 | 2 | 100 | | 136 | | 42 |
| *Phenylobacterium* | 30 | 16 | 2 | 51 | | 63 | | 42 |
| *Pseudonocardia* | 3 | 3 | 2 | 95 | | 125 | | 42 |
| *Corallococcus* | 0 | 2.3 | 1.75 | 116 | | 132 | | 43 |
| *Niastella* | 24.7 | 54.95 | 1.59 | 55 | | 32 | | 44 |
| *Herbaspirillum* | 28.27 | 7 | 1.5 | 52 | | 92 | | 45 |
| *Leifsonia* | 0 | 0.51 | 1.5 | 116 | | 172 | | 45 |
| *Rubrivivax* | 1 | 4.65 | 1.5 | 103 | | 108 | | 45 |
| *Flavisolibacter* | 45.33 | 104.1 | 1.34 | 44 | | 19 | | 46 |
| *Methylocystis* | 4.86 | 3.07 | 1.33 | 88 | | 124 | | 47 |
| *Xylophilus* | 0.03 | 0.03 | 1.25 | 114 | | 213 | | 48 |
| *Acidisphaera* | 66.19 | 16.92 | 1 | 36 | | 61 | | 49 |
| *Aquabacterium* | 1 | 0.95 | 1 | 103 | | 155 | | 49 |
| *Arenimonas* | 24 | 1 | 1 | 56 | | 154 | | 49 |
| *Azoarcus* | 33.14 | 23.96 | 1 | 49 | | 50 | | 49 |
| *Bacillariophyta* | 1 | 4 | 1 | 103 | | 115 | | 49 |
| *Balneimonas* | 9 | 1.31 | 1 | 78 | | 144 | | 49 |
| *Bdellovibrio* | 25 | 10 | 1 | 54 | | 81 | | 49 |
| BRC1_genera_incertae_sedis | 12 | 12 | 1 | 75 | | 72 | | 49 |
| *Brevibacillus* | 1 | 2 | 1 | 103 | | 136 | | 49 |
| *Catelliglobosispora* | 0 | 2 | 1 | 116 | | 136 | | 49 |
| *Chloroflexus* | 0 | 1 | 1 | 116 | | 154 | | 49 |
| *Cupriavidus* | 8 | 0.92 | 1 | 79 | | 156 | | 49 |
| *Dactylosporangium* | 7 | 7 | 1 | 82 | | 92 | | 49 |
| *Dehalogenimonas* | 2 | 61 | 1 | 100 | | 29 | | 49 |
| *Denitratisoma* | 4.05 | 0.5 | 1 | 91 | | 173 | | 49 |
| *Desulfitobacterium* | 1 | 1 | 1 | 103 | | 154 | | 49 |
| *Dokdonella* | 23 | 13 | 1 | 58 | | 66 | | 49 |
| *Enhygromyxa* | 5 | 5 | 1 | 87 | | 105 | | 49 |
| Gp11 | 13 | 19 | 1 | 73 | | 59 | | 49 |
| GpI | 0 | 0.9 | 1 | 116 | | 158 | | 49 |
| *Herpetosiphon* | 1 | 2 | 1 | 103 | | 136 | | 49 |
| *Hirschia* | 22 | 11 | 1 | 61 | | 77 | | 49 |
| *Kribbella* | 2 | 9 | 1 | 100 | | 84 | | 49 |
| *Ktedonobacter* | 16 | 1 | 1 | 68 | | 154 | | 49 |
| *Massilia* | 4 | 4 | 1 | 92 | | 115 | | 49 |
| *Nakamurella* | 0 | 0 | 1 | 116 | | 225 | | 49 |
| *Pseudomonas* | 0 | 0 | 1 | 116 | | 225 | | 49 |
| *Ramlibacter* | 43.31 | 20.5 | 1 | 46 | | 55 | | 49 |
| *Rhodococcus* | 0 | 10.42 | 1 | 116 | | 80 | | 49 |
| *Schlesneria* | 0 | 0 | 1 | 116 | | 225 | | 49 |
| *Simiduia* | 0 | 0 | 1 | 116 | | 225 | | 49 |
| *Singulisphaera* | 0 | 4 | 1 | 116 | | 115 | | 49 |
| *Staphylococcus* | 0 | 0.12 | 1 | 116 | | 199 | | 49 |
| *Sterolibacterium* | 4.7 | 4.7 | 1 | 89 | | 107 | | 49 |
| Subdivision5_genera_incertae_sedis | 0 | 0 | 1 | 116 | | 225 | | 49 |
| *Telmatospirillum* | 1 | 2 | 1 | 103 | | 136 | | 49 |
| *Tumebacillus* | 2 | 8 | 1 | 100 | | 87 | | 49 |
| *Verrucomicrobium* | 9 | 9 | 1 | 78 | | 84 | | 49 |
| *Virgisporangium* | 7 | 6 | 1 | 82 | | 97 | | 49 |
| *Methylobacillus* | 58.62 | 30.33 | 0.95 | 41 | | 43 | | 50 |
| *Sediminibacterium* | 5 | 0.17 | 0.59 | 87 | | 196 | | 51 |
| *Chitinophaga* | 0 | 4.34 | 0.58 | 116 | | 112 | | 52 |
| *Agromyces* | 0 | 0 | 0.5 | 116 | | 225 | | 53 |
| *Lysinibacillus* | 0 | 2.06 | 0.5 | 116 | | 135 | | 53 |
| *Microbacterium* | 0 | 1.16 | 0.5 | 116 | | 147 | | 53 |
| *Microbispora* | 3 | 1.93 | 0.5 | 95 | | 137 | | 53 |
| *Microtetraspora* | 0 | 0.59 | 0.5 | 116 | | 168 | | 53 |
| *Myxococcus* | 0 | 1 | 0.5 | 116 | | 154 | | 53 |
| *Nitrobacter* | 1.52 | 19.05 | 0.5 | 101 | | 58 | | 53 |
| *Planococcus* | 0 | 0 | 0.5 | 116 | | 225 | | 53 |
| *Rathayibacter* | 0 | 0 | 0.5 | 116 | | 225 | | 53 |
| *Rhodopseudomonas* | 0 | 3.33 | 0.5 | 116 | | 122 | | 53 |
| *Saccharomonospora* | 0 | 0.46 | 0.5 | 116 | | 175 | | 53 |
| *Saccharopolyspora* | 0 | 0 | 0.5 | 116 | | 225 | | 53 |
| *Sphingobium* | 0 | 3.89 | 0.5 | 116 | | 116 | | 53 |
| *Filimonas* | 2 | 1 | 0.48 | 100 | | 154 | | 54 |
| *Curvibacter* | 1.25 | 1.25 | 0.45 | 102 | | 146 | | 55 |
| *Segetibacter* | 0 | 3.83 | 0.34 | 116 | | 117 | | 56 |
| *Friedmanniella* | 0 | 0 | 0.33 | 116 | | 225 | | 57 |
| *Methylobacterium* | 9 | 5.26 | 0.33 | 78 | | 104 | | 57 |
| *Microlunatus* | 4 | 4 | 0.33 | 92 | | 115 | | 57 |
| *Patulibacter* | 0 | 0 | 0.33 | 116 | | 225 | | 57 |
| *Propionibacterium* | 0 | 0 | 0.33 | 116 | | 225 | | 57 |
| *Cystobacter* | 19 | 11.7 | 0.25 | 65 | | 75 | | 58 |
| *Hyalangium* | 10 | 5 | 0.25 | 76 | | 105 | | 58 |
| *Stigmatella* | 0 | 0 | 0.25 | 116 | | 225 | | 58 |
| *Thiomonas* | 0 | 0 | 0.25 | 116 | | 225 | | 58 |
| *Zoogloea* | 0 | 5.41 | 0.25 | 116 | | 102 | | 58 |
| *Dechloromonas* | 0 | 0.43 | 0.17 | 116 | | 177 | | 59 |
| *Acetivibrio* | 2 | 2 | 0 | 100 | | 136 | | 60 |
| *Acidicaldus* | 0 | 2.26 | 0 | 116 | | 133 | | 60 |
| *Acidimicrobium* | 18.5 | 2.5 | 0 | 66 | | 128 | | 60 |
| *Acidiphilium* | 3.14 | 0.23 | 0 | 93 | | 194 | | 60 |
| *Acidothermus* | 17 | 14.5 | 0 | 67 | | 65 | | 60 |
| *Acidovorax* | 2.84 | 2.84 | 0 | 96 | | 126 | | 60 |
| *Acrocarpospora* | 0 | 0.07 | 0 | 116 | | 205 | | 60 |
| *Actinoallomurus* | 3 | 3 | 0 | 95 | | 125 | | 60 |
| *Actinoalloteichus* | 0.67 | 1.29 | 0 | 107 | | 145 | | 60 |
| *Actinocorallia* | 1 | 1.92 | 0 | 103 | | 138 | | 60 |
| *Actinokineospora* | 5.08 | 3 | 0 | 86 | | 125 | | 60 |
| *Actinomadura* | 3 | 0.25 | 0 | 95 | | 191 | | 60 |
| *Actinoplanes* | 9.14 | 3.4 | 0 | 77 | | 121 | | 60 |
| *Adhaeribacter* | 1 | 1 | 0 | 103 | | 154 | | 60 |
| *Aeromicrobium* | 4 | 1.86 | 0 | 92 | | 139 | | 60 |
| *Afifella* | 3 | 19.55 | 0 | 95 | | 57 | | 60 |
| *Afipia* | 7.94 | 1.25 | 0 | 81 | | 146 | | 60 |
| *Agrococcus* | 0 | 0.15 | 0 | 116 | | 197 | | 60 |
| *Ahrensia* | 0 | 0.01 | 0 | 116 | | 223 | | 60 |
| *Albidovulum* | 1 | 1 | 0 | 103 | | 154 | | 60 |
| *Allokutzneria* | 0 | 0.24 | 0 | 116 | | 192 | | 60 |
| *Altererythrobacter* | 0.36 | 0.36 | 0 | 110 | | 181 | | 60 |
| *Amorphus* | 0 | 1 | 0 | 116 | | 154 | | 60 |
| *Amycolatopsis* | 1 | 3.54 | 0 | 103 | | 120 | | 60 |
| *Ancylobacter* | 2 | 0.77 | 0 | 100 | | 163 | | 60 |
| *Aneurinibacillus* | 2 | 2 | 0 | 100 | | 136 | | 60 |
| *Aquaspirillum* | 1 | 1 | 0 | 103 | | 154 | | 60 |
| *Arthrobacter* | 7 | 11.73 | 0 | 82 | | 74 | | 60 |
| *Asaia* | 0 | 0.39 | 0 | 116 | | 179 | | 60 |
| *Asanoa* | 0 | 0.11 | 0 | 116 | | 200 | | 60 |
| *Aspromonas* | 1 | 1 | 0 | 103 | | 154 | | 60 |
| *Azohydromonas* | 3 | 0.67 | 0 | 95 | | 166 | | 60 |
| *Azonexus* | 3.04 | 3.74 | 0 | 94 | | 118 | | 60 |
| *Azospirillum* | 2 | 93.88 | 0 | 100 | | 20 | | 60 |
| *Azovibrio* | 1 | 1 | 0 | 103 | | 154 | | 60 |
| *Bartonella* | 0 | 0.21 | 0 | 116 | | 195 | | 60 |
| *Beijerinckia* | 1 | 8.46 | 0 | 103 | | 86 | | 60 |
| *Blastochloris* | 0.8 | 29.74 | 0 | 106 | | 45 | | 60 |
| *Blastococcus* | 4 | 2.35 | 0 | 92 | | 130 | | 60 |
| *Bordetella* | 1 | 1 | 0 | 103 | | 154 | | 60 |
| *Brevibacterium* | 0 | 0.25 | 0 | 116 | | 191 | | 60 |
| *Brevundimonas* | 0 | 2 | 0 | 116 | | 136 | | 60 |
| *Caenimonas* | 1 | 1 | 0 | 103 | | 154 | | 60 |
| *Catellatospora* | 0.9 | 0.34 | 0 | 104 | | 185 | | 60 |
| *Catenulispora* | 0 | 1.73 | 0 | 116 | | 140 | | 60 |
| *Catenuloplanes* | 0 | 6.24 | 0 | 116 | | 95 | | 60 |
| *Caulobacter* | 12 | 6 | 0 | 75 | | 97 | | 60 |
| *Cellulomonas* | 0 | 0.48 | 0 | 116 | | 174 | | 60 |
| *Chelatococcus* | 3 | 18.37 | 0 | 95 | | 60 | | 60 |
| *Chlorophyta* | 0 | 4 | 0 | 116 | | 115 | | 60 |
| *Chondromyces* | 2.5 | 4.3 | 0 | 97 | | 114 | | 60 |
| *Citricoccus* | 0 | 0.28 | 0 | 116 | | 190 | | 60 |
| *Clostridium* | 1 | 4 | 0 | 103 | | 115 | | 60 |
| *Cohnella* | 1 | 1 | 0 | 103 | | 154 | | 60 |
| *Crocinitomix* | 3 | 3 | 0 | 95 | | 125 | | 60 |
| *Crossiella* | 0 | 1 | 0 | 116 | | 154 | | 60 |
| *Cryobacterium* | 0 | 0.07 | 0 | 116 | | 206 | | 60 |
| *Cryptosporangium* | 0 | 0.82 | 0 | 116 | | 160 | | 60 |
| *Defluviicoccus* | 0 | 1.15 | 0 | 116 | | 148 | | 60 |
| *Dermacoccus* | 0 | 0.71 | 0 | 116 | | 164 | | 60 |
| *Derxia* | 1 | 1 | 0 | 103 | | 154 | | 60 |
| *Desulfosporosinus* | 0 | 2 | 0 | 116 | | 136 | | 60 |
| *Desulfotalea* | 0 | 2 | 0 | 116 | | 136 | | 60 |
| *Desulfuromonas* | 1 | 1 | 0 | 103 | | 154 | | 60 |
| *Devosia* | 22 | 6.79 | 0 | 61 | | 94 | | 60 |
| *Duganella* | 1 | 0.5 | 0 | 103 | | 173 | | 60 |
| *Dyella* | 2 | 2 | 0 | 100 | | 136 | | 60 |
| *Ectothiorhodosinus* | 2 | 2 | 0 | 100 | | 136 | | 60 |
| *Ectothiorhodospira* | 0 | 2 | 0 | 116 | | 136 | | 60 |
| *Ensifer* | 0 | 0.03 | 0 | 116 | | 215 | | 60 |
| *Ferrimicrobium* | 5 | 4 | 0 | 87 | | 115 | | 60 |
| *Filomicrobium* | 0 | 9.3 | 0 | 116 | | 82 | | 60 |
| *Flavobacterium* | 0 | 7 | 0 | 116 | | 92 | | 60 |
| *Frigoribacterium* | 0 | 0.07 | 0 | 116 | | 206 | | 60 |
| *Frondihabitans* | 0 | 0.01 | 0 | 116 | | 222 | | 60 |
| *Geminicoccus* | 1 | 1 | 0 | 103 | | 154 | | 60 |
| *Geobacillus* | 1 | 1 | 0 | 103 | | 154 | | 60 |
| *Gluconobacter* | 0 | 0.59 | 0 | 116 | | 169 | | 60 |
| *Gordonia* | 0 | 3.21 | 0 | 116 | | 123 | | 60 |
| *Gordonibacter* | 1 | 1 | 0 | 103 | | 154 | | 60 |
| Gp15 | 0 | 2 | 0 | 116 | | 136 | | 60 |
| Gp20 | 1 | 1 | 0 | 103 | | 154 | | 60 |
| Gp25 | 46 | 20 | 0 | 43 | | 56 | | 60 |
| GpXIII | 0 | 0.1 | 0 | 116 | | 201 | | 60 |
| *Gracilibacter* | 3 | 3 | 0 | 95 | | 125 | | 60 |
| *Granulibacter* | 0 | 1 | 0 | 116 | | 154 | | 60 |
| *Haliea* | 0 | 1 | 0 | 116 | | 154 | | 60 |
| *Hamadaea* | 0 | 0.02 | 0 | 116 | | 219 | | 60 |
| *Herbidospora* | 0 | 0.01 | 0 | 116 | | 221 | | 60 |
| *Herminiimonas* | 12.73 | 12.73 | 0 | 74 | | 68 | | 60 |
| *Hoeflea* | 2 | 2 | 0 | 100 | | 136 | | 60 |
| *Humicoccus* | 5.67 | 1 | 0 | 85 | | 154 | | 60 |
| *Hydrocarboniphaga* | 1 | 1 | 0 | 103 | | 154 | | 60 |
| *Ideonella* | 6 | 12.48 | 0 | 84 | | 70 | | 60 |
| *Janibacter* | 1 | 0.05 | 0 | 103 | | 211 | | 60 |
| *Jeotgalibacillus* | 0 | 0.25 | 0 | 116 | | 191 | | 60 |
| *Kibdelosporangium* | 2 | 1 | 0 | 100 | | 154 | | 60 |
| *Kineococcus* | 0 | 4.79 | 0 | 116 | | 106 | | 60 |
| *Kineosporia* | 5 | 5.53 | 0 | 87 | | 99 | | 60 |
| *Kitasatospora* | 0 | 2.45 | 0 | 116 | | 129 | | 60 |
| *Knoellia* | 0 | 0.05 | 0 | 116 | | 208 | | 60 |
| *Kocuria* | 0 | 0.05 | 0 | 116 | | 210 | | 60 |
| *Krasilnikovia* | 1 | 5.5 | 0 | 103 | | 100 | | 60 |
| *Kurthia* | 0 | 0.91 | 0 | 116 | | 157 | | 60 |
| *Labedella* | 0 | 0.05 | 0 | 116 | | 207 | | 60 |
| *Labrys* | 6 | 1.14 | 0 | 84 | | 149 | | 60 |
| *Lechevalieria* | 0.38 | 0.38 | 0 | 109 | | 180 | | 60 |
| *Legionella* | 3 | 3 | 0 | 95 | | 125 | | 60 |
| *Lentzea* | 0.03 | 0.03 | 0 | 115 | | 214 | | 60 |
| *Leptolinea* | 0 | 7.25 | 0 | 116 | | 90 | | 60 |
| *Leptonema* | 1 | 1 | 0 | 103 | | 154 | | 60 |
| *Leptothrix* | 16 | 9.26 | 0 | 68 | | 83 | | 60 |
| *Longilinea* | 0 | 48.75 | 0 | 116 | | 34 | | 60 |
| *Longispora* | 0 | 2 | 0 | 116 | | 136 | | 60 |
| *Luedemannella* | 12 | 1.07 | 0 | 75 | | 151 | | 60 |
| *Luteimonas* | 0.5 | 1 | 0 | 108 | | 154 | | 60 |
| *Lysobacter* | 2.5 | 10.65 | 0 | 97 | | 78 | | 60 |
| *Magnetospirillum* | 0 | 21.95 | 0 | 116 | | 52 | | 60 |
| *Mesorhizobium* | 27 | 12.66 | 0 | 53 | | 69 | | 60 |
| *Methylocella* | 2 | 5.84 | 0 | 100 | | 98 | | 60 |
| *Methylosinus* | 0.14 | 0.09 | 0 | 113 | | 203 | | 60 |
| *Methyloversatilis* | 0.25 | 4.5 | 0 | 112 | | 110 | | 60 |
| *Microcella* | 0 | 0.05 | 0 | 116 | | 207 | | 60 |
| *Micrococcus* | 0 | 0.35 | 0 | 116 | | 183 | | 60 |
| *Micromonospora* | 7.96 | 12.27 | 0 | 80 | | 71 | | 60 |
| *Microvirga* | 2 | 3 | 0 | 100 | | 125 | | 60 |
| *Modestobacter* | 0 | 0.59 | 0 | 116 | | 170 | | 60 |
| *Mycetocola* | 0 | 0.15 | 0 | 116 | | 197 | | 60 |
| *Nannocystis* | 1 | 1 | 0 | 103 | | 154 | | 60 |
| *Neoasaia* | 0 | 0.02 | 0 | 116 | | 220 | | 60 |
| *Nevskia* | 1 | 1 | 0 | 103 | | 154 | | 60 |
| *Niabella* | 0 | 0.58 | 0 | 116 | | 171 | | 60 |
| *Nitriliruptor* | 0 | 3.67 | 0 | 116 | | 119 | | 60 |
| *Nocardia* | 2 | 8.53 | 0 | 100 | | 85 | | 60 |
| *Nonomuraea* | 0 | 0.8 | 0 | 116 | | 161 | | 60 |
| *Novispirillum* | 0 | 5.33 | 0 | 116 | | 103 | | 60 |
| *Novosphingobium* | 0 | 4.61 | 0 | 116 | | 109 | | 60 |
| *Oceanobacillus* | 0 | 0.32 | 0 | 116 | | 189 | | 60 |
| *Ochrobactrum* | 0 | 0.03 | 0 | 116 | | 215 | | 60 |
| *Okibacterium* | 0 | 0.09 | 0 | 116 | | 202 | | 60 |
| *Olsenella* | 0 | 0.83 | 0 | 116 | | 159 | | 60 |
| *Orientia* | 2 | 3 | 0 | 100 | | 125 | | 60 |
| *Oryzihumus* | 1 | 3 | 0 | 103 | | 125 | | 60 |
| *Oxalicibacterium* | 8 | 0.08 | 0 | 79 | | 204 | | 60 |
| *Paenibacillus* | 17 | 29 | 0 | 67 | | 46 | | 60 |
| *Parvibaculum* | 1 | 1 | 0 | 103 | | 154 | | 60 |
| *Pelobacter* | 0.83 | 0.83 | 0 | 105 | | 159 | | 60 |
| *Pelomonas* | 0 | 1 | 0 | 116 | | 154 | | 60 |
| *Phaeospirillum* | 0 | 1.67 | 0 | 116 | | 141 | | 60 |
| *Phycicoccus* | 3 | 3 | 0 | 95 | | 125 | | 60 |
| *Pilimelia* | 0 | 0.05 | 0 | 116 | | 209 | | 60 |
| *Planomonospora* | 0 | 0.04 | 0 | 116 | | 212 | | 60 |
| *Planotetraspora* | 0 | 0.34 | 0 | 116 | | 184 | | 60 |
| *Plantactinospora* | 0 | 0.33 | 0 | 116 | | 186 | | 60 |
| *Plantibacter* | 0 | 0.33 | 0 | 116 | | 187 | | 60 |
| *Pleomorphomonas* | 0 | 11.27 | 0 | 116 | | 76 | | 60 |
| *Polaromonas* | 1 | 1 | 0 | 103 | | 154 | | 60 |
| *Porphyrobacter* | 6.89 | 0.44 | 0 | 83 | | 176 | | 60 |
| *Prolixibacter* | 2 | 2 | 0 | 100 | | 136 | | 60 |
| *Promicromonospora* | 0 | 1 | 0 | 116 | | 154 | | 60 |
| *Propionivibrio* | 2.25 | 2.25 | 0 | 99 | | 134 | | 60 |
| *Pseudaminobacter* | 0 | 0.02 | 0 | 116 | | 216 | | 60 |
| *Pseudosporangium* | 0 | 0.33 | 0 | 116 | | 186 | | 60 |
| *Rhizobium* | 2 | 6.02 | 0 | 100 | | 96 | | 60 |
| *Rhodopila* | 14.33 | 12.85 | 0 | 70 | | 67 | | 60 |
| *Rickettsia* | 3 | 3 | 0 | 95 | | 125 | | 60 |
| *Roseomonas* | 2.33 | 11.91 | 0 | 98 | | 73 | | 60 |
| *Rothia* | 0 | 0.78 | 0 | 116 | | 162 | | 60 |
| *Rubellimicrobium* | 1 | 1 | 0 | 103 | | 154 | | 60 |
| *Rugosimonospora* | 1 | 1 | 0 | 103 | | 154 | | 60 |
| *Rummeliibacillus* | 0 | 1.09 | 0 | 116 | | 150 | | 60 |
| *Salinibacterium* | 0 | 0.33 | 0 | 116 | | 188 | | 60 |
| *Sandaracinobacter* | 0 | 0.02 | 0 | 116 | | 218 | | 60 |
| *Sandarakinorhabdus* | 0 | 1.01 | 0 | 116 | | 153 | | 60 |
| *Saxeibacter* | 0.67 | 0.67 | 0 | 107 | | 166 | | 60 |
| *Schumannella* | 0 | 0.02 | 0 | 116 | | 217 | | 60 |
| *Shimazuella* | 2 | 2 | 0 | 100 | | 136 | | 60 |
| *Shinella* | 0 | 0.02 | 0 | 116 | | 216 | | 60 |
| *Singularimonas* | 3 | 3 | 0 | 95 | | 125 | | 60 |
| *Skermanella* | 75 | 16.75 | 0 | 35 | | 62 | | 60 |
| *Solimonas* | 7 | 7 | 0 | 82 | | 92 | | 60 |
| *Sorangium* | 0.33 | 6 | 0 | 111 | | 97 | | 60 |
| *Sphaerisporangium* | 0 | 0.14 | 0 | 116 | | 198 | | 60 |
| *Sphaerobacter* | 14 | 1 | 0 | 71 | | 154 | | 60 |
| *Sphingosinicella* | 0 | 0.01 | 0 | 116 | | 224 | | 60 |
| *Spirillospora* | 0 | 1.33 | 0 | 116 | | 143 | | 60 |
| *Sporichthya* | 9 | 13 | 0 | 78 | | 66 | | 60 |
| *Sporocytophaga* | 22 | 15 | 0 | 61 | | 64 | | 60 |
| *Sporomusa* | 22 | 9 | 0 | 61 | | 84 | | 60 |
| *Stappia* | 0 | 2.62 | 0 | 116 | | 127 | | 60 |
| *Starkeya* | 0 | 0.23 | 0 | 116 | | 193 | | 60 |
| *Streptacidiphilus* | 0 | 0.69 | 0 | 116 | | 165 | | 60 |
| *Streptomyces* | 22.53 | 38.04 | 0 | 59 | | 38 | | 60 |
| *Streptosporangium* | 0 | 0.07 | 0 | 116 | | 206 | | 60 |
| *Syntrophobacter* | 1 | 1 | 0 | 103 | | 154 | | 60 |
| *Tatlockia* | 2 | 2 | 0 | 100 | | 136 | | 60 |
| *Tepidimonas* | 0 | 2 | 0 | 116 | | 136 | | 60 |
| *Terrabacter* | 1 | 0.65 | 0 | 103 | | 167 | | 60 |
| *Tetrasphaera* | 0 | 4.4 | 0 | 116 | | 111 | | 60 |
| *Thauera* | 4.3 | 4.3 | 0 | 90 | | 113 | | 60 |
| *Thermincola* | 7 | 1 | 0 | 82 | | 154 | | 60 |
| *Thermobacillus* | 0 | 1 | 0 | 116 | | 154 | | 60 |
| *Thermocrispum* | 3 | 3 | 0 | 95 | | 125 | | 60 |
| *Thermoflavimicrobium* | 1 | 1 | 0 | 103 | | 154 | | 60 |
| *Thermomonas* | 0 | 0.35 | 0 | 116 | | 182 | | 60 |
| *Tsukamurella* | 0 | 1 | 0 | 116 | | 154 | | 60 |
| *Tuberibacillus* | 0 | 1 | 0 | 116 | | 154 | | 60 |
| *Uliginosibacterium* | 0 | 21.42 | 0 | 116 | | 54 | | 60 |
| *Undibacterium* | 3 | 1.5 | 0 | 95 | | 142 | | 60 |
| *Ureibacillus* | 1 | 1 | 0 | 103 | | 154 | | 60 |
| *Verrucosispora* | 0 | 0.42 | 0 | 116 | | 178 | | 60 |
| *Zhihengliuella* | 0 | 0.01 | 0 | 116 | | 223 | | 60 |
| *Zimmermannella* | 0 | 0.01 | 0 | 116 | | 222 | | 60 |
| Total | 20,431 | 9,956 | 1,041 |  | |  | |  |

*^a^* Each value of abundance is rounded off to two decimal places.

*^b^* Rank abundances in each experiment are calculated before rounding off the values of abundance. When multiple genera have the identical values of abundance, the rank abundances of these genera become identical, and the next rank abundance is only one increment from previous rank abundance despite the number of genera with identical values of abundance.
